# Smoke characteristics of conveyor-belt fires in U-shaped belt-conveyor roadways under different ventilation velocities

**DOI:** 10.1371/journal.pone.0352936

**Published:** 2026-10-14

**Authors:** Jian Ding, Jinzhong Wu

**Affiliations:** Xi'an University of Science and Technology, Xi'an, Shaanxi, China; Henan Polytechnic University, CHINA

## Abstract

To elucidate the smoke characteristics of conveyor-belt fires in U-shaped belt-conveyor roadways under different ventilation velocities, small-scale combustion experiments were conducted using PVG1250S flame-retardant conveyor-belt specimens. A PyroSim model of a U-shaped roadway was subsequently developed to examine smoke transport under different ventilation velocities and prescribed thermal-source intensities. The results show that, as the ventilation velocity increased from 0.4 to 1.6 m/s, the peak CO concentration at the monitoring point decreased from 453 to 175 ppm, whereas the peak flow-rate-corrected CO transport index increased by 54.43%. The fixed-point temperature response was non-monotonic and reflected the combined effects of combustion fluctuation, smoke-layer position, buoyancy, and convective transport. Numerical simulations further reveal that buoyancy dominates smoke movement under low-velocity ventilation, promoting upstream back-layering of hot smoke along the roof and local accumulation of heat and CO near the fire source. As the ventilation velocity increases, the longitudinal airflow increasingly suppresses and entrains the fire plume, thereby weakening upstream back-layering while enhancing smoke transport toward the downstream roadway, working face and connecting roadway. Based on the composite smoke-response index, the scenario-specific characteristic transition velocity associated with a marked reduction in long-distance upstream smoke back-layering was approximately 2.24 m/s, with a transition range of 2.0–2.4 m/s under the prescribed 4800 kW thermal-source sensitivity scenario. The results provide a reference for understanding smoke redistribution and ventilation effects in U-shaped belt-conveyor roadways under comparable source, geometric, and ventilation conditions.

## Introduction

With increasing mining depth, haulage distance and production intensity in coal mines, belt-conveyor systems have become increasingly critical to continuous underground production. Belt-conveyor roadways serve multiple functions, including coal transport, ventilation supply and personnel inspection. Once a conveyor belt fire occurs, hot smoke and toxic or hazardous gases such as CO, CO_2_ and HCl can spread rapidly under the action of ventilation airflow, severely degrading the underground environment for evacuation and emergency rescue [1–3]. The fire hazard of mining conveyor belts is governed not only by the flame-retardant performance of the belt material [4], but also by the composition of the cover rubber, belt structure, wear condition, pyrolysis process and release characteristics of smoke products [5,6]. In semi-enclosed mine spaces, CO dispersion, thermal-pressure disturbances and ventilation-network coupling can further alter smoke propagation pathways and personnel exposure risks [7,8]. Meanwhile, the ventilation system is not only essential for maintaining normal production, but also serves as a key means of controlling the direction and rate of smoke transport during fires [9,10]. In 2020, a major fire occurred in the belt-conveyor inclined roadway of the Songzao coal mine in Chongqing, China. The accident was triggered by a jammed idler, which generated frictional heat between the idler and the belt, ignited accumulated coal, and produced rapidly spreading hot toxic smoke, resulting in severe casualties [11]. In 2023, a belt fire occurred in the haulage roadway of the Shanjiaoshu coal mine in Guizhou Province, China. Continuous friction among a jammed idler, supporting frame and conveyor belt caused temperature rise and ignited coal dust as well as a non-flame-retardant belt, leading to fatal poisoning of personnel located downstream of the fire source [12]. Therefore, investigating smoke temperature, toxic gas concentrations and their transport characteristics in belt-conveyor roadway fires under different ventilation velocities is of great significance for clarifying disaster-evolution mechanisms, optimizing ventilation-control parameters and improving emergency response capability.

In recent years, studies on smoke transport characteristics in roadway fires have mainly focused on scaled experiments and full-scale tests [13,14]. By using reduced-scale roadways, experimental roadways and full-scale roadway platforms, previous studies have investigated the diffusion of hot smoke, smoke back-layering, ceiling temperature attenuation and hazardous gas migration under varying ventilation velocities, heat release rates, fire-source locations and cross-sectional conditions [15,16]. Liang et al.[17] investigated the smoke back-layering length of double-source tunnel fires and showed that fire-source characteristics and ventilation conditions can significantly influence smoke back-layering behavior. Salami et al.[18] combined experiments with large-eddy simulation to analyze temperature attenuation, maximum excess gas temperature and gas evolution in underground mine fires, demonstrating that ventilation conditions exert a pronounced influence on temperature decay and CO transport. Further studies have shown that smoke propagation in roadway fires is not governed by a single fire-source parameter, but is jointly controlled by ventilation conditions, spatial scale, fire-source location and the release characteristics of combustion products [19]. However, existing experiments have mostly been conducted in straight roadways or local single-roadway configurations, whereas insufficient attention has been paid to smoke bifurcation, recirculation and localized accumulation in connecting roadways, turning sections and return-air regions of U-shaped transport systems.

With the development of computational fluid dynamics, numerical simulation has increasingly become an important tool for investigating smoke transport in mine fires [20,21]. Software packages such as FDS and PyroSim can resolve buoyancy-driven flow, heat transfer, smoke dispersion and species transport during fire development, thereby providing an effective approach for analyzing temperature, velocity and concentration fields in complex roadway configurations. For smoke control under longitudinal ventilation, Yao et al.[22] investigated the critical velocity and driving-force variation required to suppress smoke back-layering in coal-mine connection-roadway fires, and identified critical velocity as a key parameter for controlling upstream smoke spread. Su et al.[23] further analyzed the effects of longitudinal ventilation, critical velocity and driving force on smoke control in cross-passage configurations. Huang et al.[24] conducted reduced-scale experiments on branched tunnel fires and examined the effects of longitudinal ventilation, heat release rate and branch geometry on smoke back-layering length and critical velocity, establishing a corresponding predictive model for critical velocity. In terms of temperature-field evolution, previous studies have focused on the maximum ceiling smoke temperature, temperature attenuation and cross-sectional ventilation effects, revealing the influences of heat release rate, roadway height and ventilation conditions on hot-smoke distribution [25,26]. Moreover, several studies have extended the simulation domain from a single straight roadway to roadways with different cross-sections, branched tunnels and coal-mine connection-roadway structures, demonstrating that roadway width, bifurcation geometry and fire-source location can substantially alter smoke morphology, critical velocity and hazardous-zone distribution [27–29]. These findings indicate that smoke propagation in mine roadway fires is essentially governed by the coupled effects of ventilation conditions, roadway geometry and fire-source characteristics. Therefore, results derived solely from straight-roadway configurations or single-point measurements are insufficient to fully characterize the multi-path smoke transport behavior in U-shaped belt-conveyor roadway fires. Unlike a straight roadway, a U-shaped system forces smoke to undergo successive turning and redistribution among the belt-conveyor roadway, working face, connecting roadway, and return-air roadway. Local pressure differences, shear entrainment, and recirculation near the turning and connecting regions can consequently modify smoke bifurcation, stratification, local accumulation, and downstream transport.

Taken together, existing studies have made substantial progress in characterizing conveyor-belt combustion behavior, roadway fire experiments and smoke-transport simulations. However, the coupled evolution of temperature and CO under different ventilation velocities in U-shaped belt-conveyor roadways remains insufficiently understood. Under actual fire-disaster conditions, low ventilation velocity may promote the accumulation of hot smoke and toxic gases near the fire source, whereas excessive ventilation may accelerate the advance of the smoke front toward connecting roadways and return-air areas, thereby shortening the available evacuation time for personnel located downstream. Therefore, assessing fire hazard solely on the basis of a single temperature peak or gas concentration at an individual monitoring point is inadequate for determining appropriate ventilation-control conditions.

In this study, small-scale combustion experiments were conducted using PVG1250S flame-retardant conveyor-belt specimens to examine the temporal responses of temperature, CO, and CO_2_ under different ventilation velocities. An experimental-scale PyroSim model was developed and evaluated against the measured temperature and CO data, after which a full-scale U-shaped belt-conveyor roadway model was established to investigate smoke-flow pathways, temperature evolution, hazardous-gas transport, and smoke distribution under different ventilation velocities and prescribed thermal-source levels. Temperature and CO responses at representative monitoring locations were further analyzed to quantify the redistribution of smoke between the upstream and downstream regions and to characterize the transition in remote-upstream smoke back-layering with increasing ventilation velocity. The study focuses on the coupled effects of fire-induced buoyancy and longitudinal ventilation on smoke transport in a U-shaped roadway configuration.

## Materials and methods

### Small-scale conveyor-belt combustion experimental platform

To investigate the effects of ventilation velocity on smoke temperature and gas-concentration responses, a small-scale conveyor-belt combustion experimental platform was constructed. The system mainly consisted of a roadway model, fan, fire-source device, smoke measurement system, temperature measurement system and data acquisition system. The roadway model was 8.0 m long, 0.6 m wide, and 0.5 m high, with an arched roof radius of 0.3 m, a longitudinal slope of 0°, and 5 mm thick steel walls. A 1.5 kW variable-speed fan provided ventilation velocities of 0.4, 0.8, 1.2, and 1.6 m/s, the ambient temperature was 13°C. The gas and temperature monitoring systems were used to measure the temporal variations in CO and CO_2_ concentrations and temperature within the roadway, while the data-acquisition system continuously recorded the measured signals. The experimental platform and monitoring-point arrangement are shown in Fig 1.

**Fig 1:**
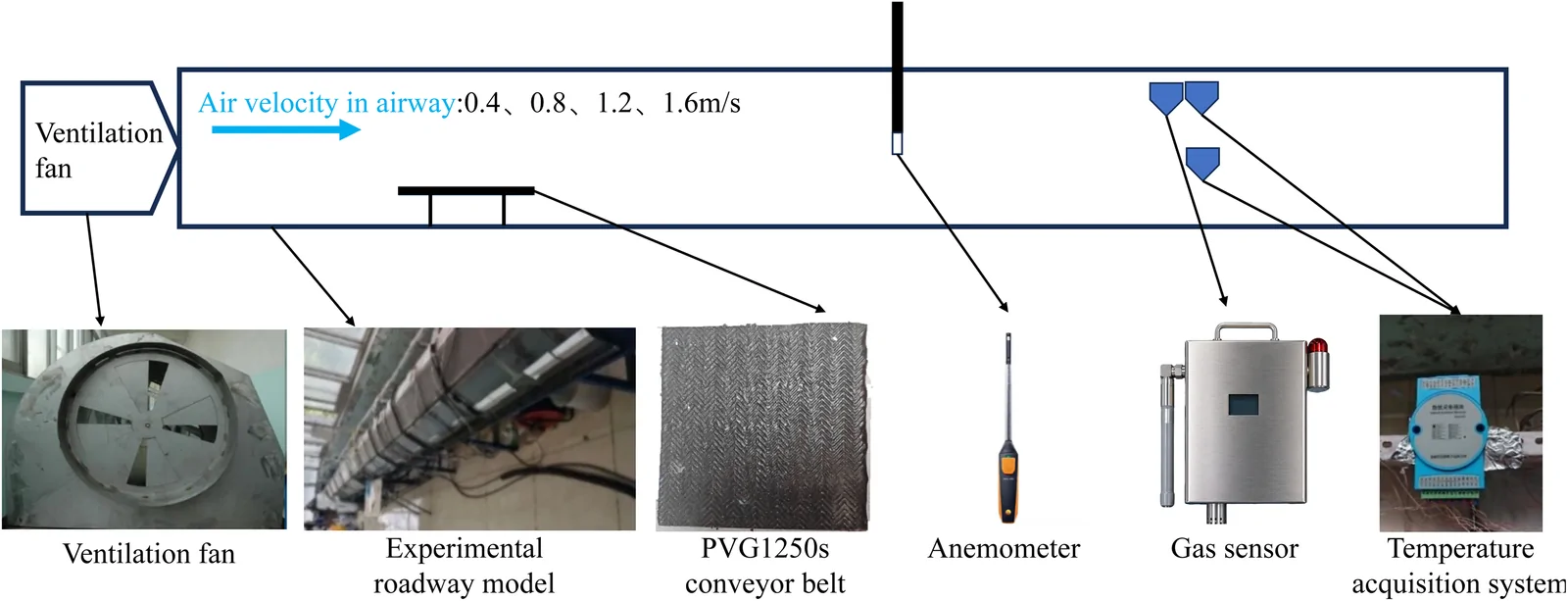
Small-scale conveyor-belt combustion experimental platform and monitoring-point arrangement.

### Experimental materials and methods

A PVG1250S flame-retardant conveyor belt used in a coal mine was selected as the experimental material in this study, with its model parameters referenced to the *Technical Standard for Belt Conveyor Engineering* (GB 50431–2020) [30]. The specimen was principally composed of PVC and nitrile rubber. The conveyor belt was cut into specimens with planar dimensions of 0.10 m × 0.10 m, a thickness of 10 mm, and a mass of 122.3 g. The specimen thickness was consistent with that of the original conveyor belt. The geometric scale ratio was defined using the characteristic cross-sectional dimensions of the roadway, with *λ* = *H_m_/H_p_* *= W_m_/W_p_* = 0.1. Froude-based scaling was used to establish the relationship between the thermal-source strengths of the reduced-scale and prototype-scale models [31]. The Froude number and the corresponding heat-release-rate scaling relationship are expressed as follows:


$$\begin{array}{c} Fr=\frac{u}{\sqrt{gL}} \end{array}$$ (1)

$$\begin{array}{c} \frac{Q_{m}}{Q_{p}}=\lambda^{\frac{\text{5}}{\text{2}}} \end{array}$$ (2)

where *Fr* is the Froude number; *u* is the characteristic longitudinal ventilation velocity m/s; *g* is the gravitational acceleration, taken as 9.81 m/s^2^, *L* is the characteristic roadway length, m; λ = H_m_/H_p_ = W_m_/W_p_ is the geometric scale ratio based on the characteristic roadway height and width; *Q* is the heat release rate, kW; and the subscripts *m* and *p* denote the reduced-scale model and prototype scale, respectively.

The release characteristics of CO and CO_2_ depend on material composition, specimen thickness, pyrolysis behavior, and local oxygen supply [5,6,32]. Accordingly, the measured concentration histories were used to characterize the local gas response of the PVG1250S specimen under the specified experimental conditions and subsequently to constrain the species-source parameters used in the numerical model.

Smoke temperature and the local CO and CO_2_ concentrations were continuously monitored under the different ventilation conditions. The combustion position was located 1.5 m from the roadway inlet. A 0.15 m × 0.15 m × 0.10 m oil pan was placed beneath a wire mesh, and the stationary single-layer specimen was positioned directly on the mesh. Industrial alcohol was used for auxiliary heating and ignition. After ignition, the ventilation velocity was adjusted to 0.4, 0.8, 1.2, or 1.6 m/s, and measurements continued until the combustion process ended. The gas sensors sampled at intervals of 0.5 s, and temperature data were recorded at intervals of 1 s. The stored gas-data intervals were 1 s for the 0.4 and 0.8 m/s cases and 10 s for the 1.2 and 1.6 m/s cases. Taking the center of the inlet floor as the coordinate origin, the gas sensors and upper thermocouple were installed at (X, Y, Z) = (5.5, 0, 0.45) m, and the lower thermocouple was installed at (5.5, 0, 0.16) m. The principal parameters of the experimental platform and monitoring system are summarized in Table 1.

**Table 1:** Principal parameters of the experimental platform and monitoring system.

Item	Parameter
Experimental roadway	8.0 m × 0.6 m × 0.5 m; arched roof radius 0.3 m; slope 0°; 5 mm steel walls
Ventilation system	1.5 kW variable-speed fan; adjustable range 0–4 m/s; test velocities 0.4, 0.8, 1.2, and 1.6 m/s
Conveyor-belt specimen	PVG1250S; principally PVC and nitrile rubber; 0.10 m × 0.10 m × 0.01 m; 122.3 g; stationary single-layer specimen
Combustion arrangement	1.5 m from inlet; oil pan 0.15 m × 0.15 m × 0.10 m; wire-mesh support; industrial alcohol for auxiliary heating and ignition
Temperature measurement	K-type surface thermocouples; −20–400°C; upper point (5.5, 0, 0.45) m; lower point (5.5, 0, 0.16) m
Gas measurement	CO: electrochemical, 0–1000 ppm, ± 5 ppm; CO_2_: infrared, 0–30%, ± 2% FS; standard-gas calibration; point (5.5, 0, 0.45) m
Air-velocity measurement	testo 405i; 0–30 m/s; accuracy ±(0.1 m/s + 5% of measured value) within 0–2 m/s

Each ventilation condition was tested three times, and the corresponding time-series data were averaged. The upper- and lower-layer temperature peaks, CO and CO_2_ concentration peaks, and their occurrence times were extracted from the mean curves. Comparisons among ventilation conditions were based on the peak characteristics, temporal evolution, and relative differences in the measured responses.

The roadway cross-sectional area calculated from the arched geometry was 0.2614 m^2^. To distinguish the effect of ventilation dilution from the convective transport response at the fixed monitoring point, the peak flow-rate-corrected CO transport index was defined as:


$$\begin{array}{c} I_{CO}=u\cdot A\cdot C_{CO}\times 10^{-3} \end{array}$$ (3)

where *I*_*CO*_ is the peak flow-rate-corrected CO transport index,L/s; *u* is the mean roadway velocity m/s; *A* is the roadway cross-sectional area,m^2^; *C*_*CO*_ is the peak CO concentration at the monitoring point, ppm; and 10^−^^3^ is the unit-conversion factor. With A treated as constant and the velocity and concentration errors assumed to be independent, the relative uncertainty of I_CO_ was estimated from the stated instrument accuracies using Eq (4) [33].


$$\begin{array}{c} \frac{\delta I_{CD}}{I_{CO}}=\sqrt{{\left(\frac{\delta u}{u}\right)}^{\text{2}}+{\left(\frac{\delta C_{CO}}{C_{CO}}\right)}^{\text{2}}} \end{array}$$ (4)

where u is the mean roadway velocity,m/s; A is the roadway cross-sectional area,m^2^; and C_CO_ is the measured CO volume fraction.

## Experimental results

Fig 2A-2D shows the evolution of CO and CO_2_ concentrations during the combustion of the PVG1250S flame-retardant mining conveyor belt under different ventilation velocities. Overall, the concentrations of both gases first increased and then decreased under all conditions, indicating that the conveyor belt underwent sequential stages of heating, polymer pyrolysis, volatile release, enhanced gas-phase oxidation and subsequent depletion of combustible components. At a ventilation velocity of 0.4 m/s, the peak CO and CO_2_ concentrations reached 453 ppm and 2.39%, respectively. Under low-velocity ventilation, the relatively weak smoke-removal capacity prolonged the residence of combustion products near the monitoring point. Heating of PVC, nitrile rubber, and plasticizer components releases volatile products, while PVC dehydrochlorination produces conjugated polyene structures, aromatic products, and carbonaceous residues [34]. The high fixed-point CO response therefore reflects the combined effects of product release and local smoke accumulation. At 0.8 m/s, both concentration peaks decreased as the stronger airflow enhanced smoke transport and dilution at the monitoring position. At 1.2 m/s, the CO peak decreased to 204 ppm, whereas the CO_2_ peak was 2.17%. This non-monotonic fixed-point response was associated with changes in product release, smoke-layer position, longitudinal transport, and ventilation dilution. Ventilation velocity can also affect flame spread, heat release rate, and the generation and transport of CO and CO_2_ during conveyor-belt fires [35]. At 1.6 m/s, the peak CO and CO_2_ concentrations were 175 ppm and 1.33%, respectively. The upper-point concentrations were jointly affected by product release, vertical stratification, longitudinal transport, and ventilation dilution [36]; their variations therefore characterize the coupled source–transport response at the monitoring location. Therefore, the differences in CO and CO_2_ concentrations under different ventilation velocities mainly arise from the coupled effects of PVG1250S conveyor-belt pyrolysis kinetics, gas-phase oxidation, surface heat feedback, and airflow-driven heat and mass transfer. At ventilation velocities of 0.4, 0.8, 1.2, and 1.6 m/s, the nominal peak flow-rate-corrected CO transport indices, expressed in L/s, were 0.0474, 0.0572, 0.0640, and 0.0732, respectively. Thus, although the local CO peak decreased by 61.37%, the corrected index increased by 54.43%, indicating that stronger dilution at a fixed point can coexist with enhanced downstream convective transport. Because the index combines the upper-point concentration with the mean roadway flow rate, it characterizes the relative transport response among the four cases. The corresponding peak concentrations, corrected indices, and estimated uncertainties are summarized in Table 2.

**Table 2:** Peak CO concentrations and peak flow-rate-corrected CO transport indices with propagated instrumental uncertainty estimates.

Velocity (m/s)	Peak CO (ppm)	I_CO_ (L/s)
0.4	453	0.0474 ± 0.0142
0.8	273.49	0.0572 ± 0.0101
1.2	204	0.0640 ± 0.0087
1.6	175	0.0732 ± 0.0085

**Fig 2:**
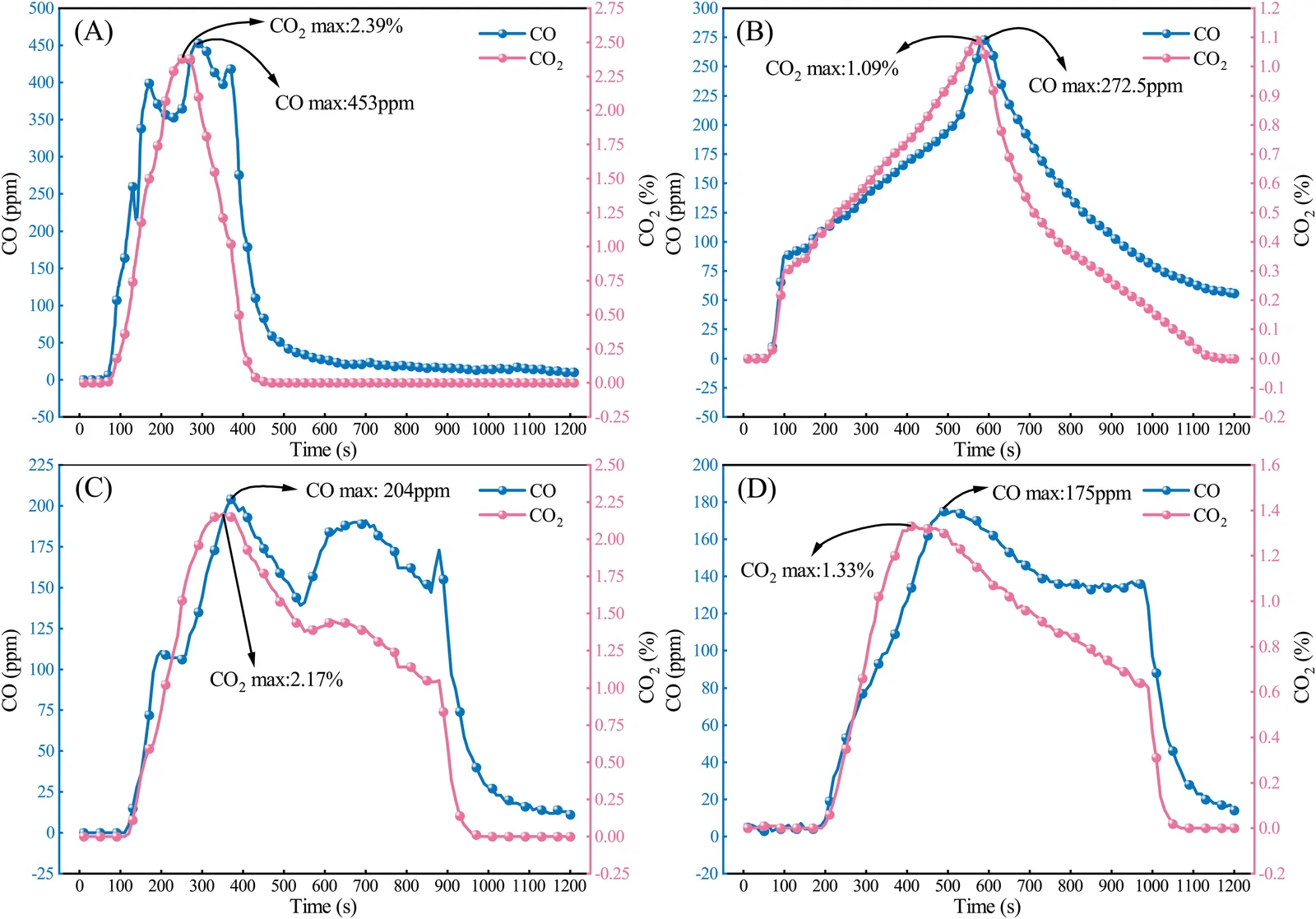
Evolution of CO and CO_2_ concentrations in the roadway under different ventilation velocities. (A) 0.4 m/s. (B) 0.8 m/s. (C) 1.2 m/s. (D) 1.6 m/s.

Fig 3A-3D presents the temperature evolution in the roadway during the combustion of the PVG1250S flame-retardant mining conveyor belt under different ventilation velocities. Overall, the upper-layer temperature was markedly higher than the lower-layer temperature under all conditions, indicating that the hot smoke released by combustion accumulated in the upper part of the roadway under buoyancy, forming a pronounced vertical thermal stratification. At a ventilation velocity of 0.4 m/s, the upper- and lower-layer temperature peaks were 97.9°C and 60.0°C, respectively. The temperature rose earlier and reached relatively high peak values, suggesting that heat released near the fire source was not readily removed under low-velocity ventilation. Strong surface heat feedback promoted the sustained pyrolysis of organic components in the PVG conveyor belt, including PVC, nitrile rubber and plasticizers. The released volatiles subsequently mixed with air, underwent gas-phase combustion and released heat [36]. At 0.8 m/s, the upper- and lower-layer temperature peaks were 99.3°C and 58.4°C, respectively. The slightly higher upper-layer peak than that at 0.4 m/s reflected the combined effects of combustion fluctuation, smoke-layer position, and convective transport, while the hot smoke remained concentrated in the upper roadway. At 1.2 m/s, the upper- and lower-layer temperature peaks decreased to 78.3°C and 48.1°C, respectively, indicating stronger convective heat removal and cold-air replenishment at the monitoring section. At 1.6 m/s, the upper- and lower-layer temperature peaks were 87.7°C and 36.1°C, respectively. The continued decrease in the lower-layer peak indicates stronger cold-air replenishment and forced-convection cooling in the lower roadway, while the upper hot-smoke layer remained evident. In conveyor-belt fires, ventilation velocity can influence flame spread, heat release rate and smoke transport [37]. To quantify vertical thermal stratification, the simultaneous upper–lower temperature difference and its maximum over the test duration were defined using Eqs (5) and (6), where *T*_*u*_*(t)* and *T*_*l*_*(t)* are the synchronous upper- and lower-layer temperatures. The maximum vertical temperature differences at 0.4, 0.8, 1.2, and 1.6 m/s were 51.14, 59.96, 52.47, and 52.97°C, respectively. The largest value occurred at 0.8 m/s, indicating that the upper hot-smoke layer reached the monitoring section while the lower region remained relatively cool. The persistent temperature separation among all cases confirms that buoyancy-driven stratification was maintained despite changes in longitudinal ventilation.


$$\begin{array}{c} \Delta T\;\left(t\right)=T_{u}\left(t\right)-T_{l}\left(t\right) \end{array}$$ (5)

$$\begin{array}{c} \Delta T_{max}=max\left[\Delta T(t)\right] \end{array}$$ (6)

**Fig 3:**
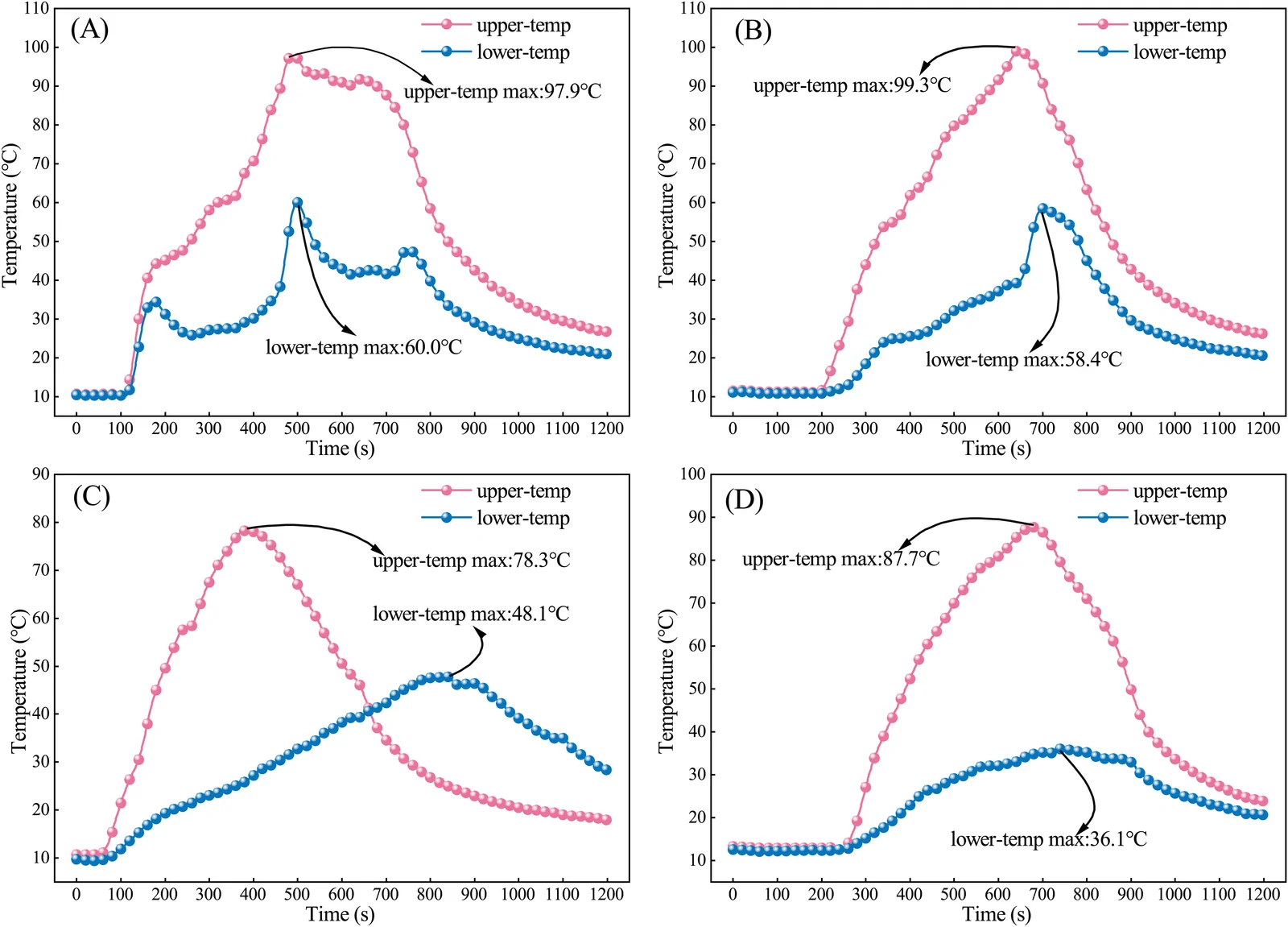
Evolution of roadway temperature under different ventilation velocities. (A) 0.4 m/s. (B) 0.8 m/s. (C) 1.2 m/s. (D) 1.6 m/s.

Therefore, the differences in temperature response under varying ventilation velocities are jointly controlled by pyrolytic heat release, gas-phase combustion intensity, surface heat feedback, buoyancy-induced stratification, and airflow-driven heat and mass transfer.

## Numerical modelling of smoke transport in a U-shaped belt-conveyor roadway under prescribed thermal-source conditions

### Theoretical framework of the numerical model

Two numerical models were established in PyroSim. The experimental-scale model reproduced the experimental geometry, fire-source position, monitoring arrangement, and ventilation conditions, and its temperature and CO responses were evaluated against the measurements obtained at 0.8 and 1.6 m/s. On this basis, an actual-scale U-shaped roadway model was further established to investigate smoke transport through the belt-conveyor roadway, working face, connecting roadway, and return-air roadway under prescribed thermal-source and ventilation conditions. For the 0.8 m/s case, the inlet velocity in the numerical model was set to 0.8 m/s to enable direct comparison between the simulation results and experimental data under identical ventilation conditions. In belt-conveyor roadway fires, the PVG1250S conveyor belt undergoes pyrolysis upon heating and releases combustible volatile products. After mixing with air, these volatiles burn to generate smoke species such as CO and CO_2_ while releasing heat. The heated gases near the fire source expand and form a buoyant plume, whereas external ventilation further affects the migration, dilution and stratification of hot smoke [38]. Therefore, the evolution of the temperature and gas concentration fields within the roadway can be described by the conservation equations of mass, momentum and energy, together with the species transport equation, as follows:


$$\begin{array}{c} \frac{\partial \rho }{\partial t}+\nabla \cdot \left(\rho 𝐮\right)=0 \end{array}$$ (7)

$$\begin{array}{c} \frac{\partial \rho u}{\partial t}+\nabla \cdot \left(\rho uu\right)=-\nabla p+\rho g+\nabla \cdot \tau \end{array}$$ (8)

$$\begin{array}{c} \frac{\partial \rho h}{\partial t}+\nabla \cdot \left(\rho h𝐮\right)=\frac{Dp}{Dt}+{\dot{q}}^{{\;}^{\prime \prime }}-\nabla \cdot 𝐪 \end{array}$$ (9)

$$\begin{array}{c} \frac{\partial \rho Y_{i}}{\partial t}+\nabla \cdot \left(\rho 𝐮Y_{i}\right)=\nabla \cdot (\rho D_{i}\nabla Y_{i})+{\dot{m}}_{i}^{{\;}^{\prime \prime \prime }} \end{array}$$ (10)

Where ρ is the gas density, kg/m^3^; *u* is the velocity vector, m/s; *t* is time, s; *p* is pressure, Pa; *g* is the gravita*t*ional acceleration,m/s^2^; *τ*is the viscous stress tensor; *h* is the *g*as enthalpy, J/kg; ${\dot{q}}^{{\;}^{\prime \prime }}$ is the volumetric heat release rate, W/m^3^; *q* is the heat flux, W/m^2^; *Y*_*i*_ is the mass fraction of the i-th gas species; *D*_*i*_ is the diffusion coefficient, m^2^/s; ${\dot{m}}_{i}^{{\;}^{\prime \prime \prime }}$ is the generation or consumption rate of the i-th species, kg/(m^3^·s).

In the actual-scale U-shaped roadway model, the fire region was represented by a fixed-area equivalent surface source of 1.0 m × 1.0 m. Using a scenario-based design-fire framework for conveyor-belt fire analysis [39], 2400 kW was prescribed as the baseline thermal-source level for the comparative analysis. With a geometric scale ratio of λ = 0.1, the corresponding reduced-scale source dimensions are 0.10 m × 0.10 m, consistent with the planar dimensions of the conveyor-belt specimen used in the experiment.

According to the Froude similarity relationship given in Eq. (2) [31], the heat-release-rate scaling factor corresponding to the geometric scale ratio λ = 0.1 is


$$\begin{array}{c} S_{Q}=\lambda^{5/2}=3.162\times 10^{-3} \end{array}$$ (11)

For the prescribed prototype-scale baseline thermal-source level of 2400 kW, the corresponding reduced-scale equivalent heat release rate is


$$\begin{array}{c} {\overset{•}{Q}}_{m,B}={\overset{•}{Q}}_{p,B}S_{Q}=2400\times 3.126\times 10^{-3}\approx 7.59\text{kW} \end{array}$$ (12)

For the 1.0 m^2^ equivalent source area in the actual-scale model, the corresponding baseline heat release rate per unit area is


$$\begin{array}{c} HRRPUA_{B}=\frac{{\overset{•}{Q}}_{p,B}}{A_{f}}=2400kW/m^{\text{2}} \end{array}$$ (13)

A second thermal-source level was specified at twice the baseline value:


$$\begin{array}{c} {\overset{•}{Q}}_{p,H}=2{\overset{•}{Q}}_{p,B}=4800kW \end{array}$$ (14)

where ${\overset{•}{Q}}_{m,B}$ and ${\overset{•}{Q}}_{p,B}$ denote the reduced-scale and prototype-scale baseline equivalent heat release rates, respectively; $S_{Q}$ is the heat-release-rate scaling factor; $A_{f}$ is the equivalent source area; and the subscripts B and H denote the baseline and higher thermal-source levels, respectively.

The resulting 7.59 kW source strength was applied in the experimental-scale model. The temperature and CO histories obtained at ventilation velocities of 0.8 and 1.6 m/s were then compared with the experimental measurements in terms of peak magnitude, peak occurrence time, and overall temporal evolution. Experimental–numerical comparison of transient thermal and species-transport responses has also been adopted in previous underground-mine fire modelling studies [40]. The agreement obtained in the present comparisons supports the use of the corresponding equivalent-source representation in the subsequent smoke-transport analysis.

The 2400 and 4800 kW cases were used as two prescribed thermal-source levels under identical source geometry and ventilation boundaries. The 4800 kW case provides a twofold thermal-forcing condition relative to the baseline scenario, allowing the response of the smoke field to thermal-source intensity to be examined within the same roadway configuration. The use of prescribed heat-release-rate levels for comparative scenario analysis is consistent with the design-fire approach adopted in conveyor-belt fire studies [41].

After the thermal-source levels had been specified, the CO and CO_2_ mass fluxes and time functions were calibrated using the measured concentration histories. During this procedure, the source area, source position, and thermal-source intensity were fixed, while the species-source terms were adjusted to reproduce the peak levels and overall temporal evolution of the measured gas concentrations. Under ventilation, the gases released from the fire source were transported downstream with the airflow. To obtain an initial reference scale for the species-source calibration, an apparent unit-area species flux was estimated from the measured local concentration response and the roadway ventilation flow as follows:


$$\begin{array}{c} {\overset{•}{m}}_{i}\left(t\right)=\frac{\rho uA}{A_{f}}X_{i}\left(t\right)\frac{M_{i}}{M_{a}} \end{array}$$ (15)

Where ${\overset{•}{m}}_{i}\left(t\right)$ is the apparent unit-area species flux of species *i*, kg/(m^2^·s), $A_{f}$ is the fire-source area, m^2^;$X_{i}\left(t\right)$ is the volume fraction of species i;*M*_*i*_ is the molar mass of species i; and *M*_*a*_ is the mean molar mass of air, taken as 28.97 kg/kmol.

Because the CO and CO_2_ concentrations exhibited pronounced temporal variations during combustion, the gas release process was described in this study using a combination of a reference mass flux and a time-dependent function:


$$\begin{array}{c} m_{i}\left(t\right)=m_{i,0}f_{i}\left(t\right) \end{array}$$ (16)

Where $m_{i,0}$ is the reference mass flux of species i, kg/(m^2^·s); $f_{i}\left(t\right)$is the dimensionless time-dependent function for the corresponding gas species. Independent time functions were adopted for CO and CO_2_ to account for their different release behaviors during the growth, peak and decay stages of combustion.

The apparent species flux estimated from Eq. (15) was used as the initial reference for species-source calibration. The reference mass fluxes and time-dependent functions of CO and CO_2_ were then adjusted by comparing the simulated concentration histories with the experimental measurements. The resulting reference mass fluxes of CO and CO_2_ were 9.5 × 10^−4^ kg/(m^2^·s) and 2.9 × 10^−2^ kg/(m^2^·s), respectively. In addition, the CO yield, soot yield, and radiative fraction in the equivalent-fuel combustion model were set to 0.032, 0.060, and 0.35, respectively. The experimentally constrained CO and CO_2_ reference mass fluxes and time-dependent functions define the prescribed species-release profiles, whereas the yield and radiative parameters characterize the equivalent combustion model. These parameters were used consistently across all ventilation cases and were retained for both prescribed thermal-source levels. The thermal-source scaling and species-source calibration therefore provide complementary descriptions of the thermal and combustion-product source terms in the numerical model.

The fire characteristic diameter D* calculated using Eq. (17) was used as a reference scale for evaluating the computational mesh.


$$\begin{array}{c} D^{*}={\left(\frac{\overset{•}{Q}}{\rho C_{p}T_{\alpha }\sqrt{g}}\right)}^{\frac{\text{2}}{\text{5}}} \end{array}$$ (17)

Where D* is the fire characteristic diameter, m; $\overset{•}{Q}$is the heat release rate of the fire source, kW;ρ is the air density, kg/m^3^; $C_{p}$ is the specific heat capacity at constant pressure, J/(kg/K); $T_{\alpha }$ is the ambient temperature, K; *g* is the gravitational acceleration, taken as 9.81 m/s^2^.

According to Eq (17), the fire characteristic diameters corresponding to heat release rates of 2400 and 4800 kW were 1.378 and 1.818 m, respectively. To eliminate the influence of mesh size on the numerical results, the ventilation velocity of 0.8 m/s was selected as the baseline condition for mesh-independence verification. Simulations were conducted using mesh sizes of 0.1, 0.2, 0.3, 0.4 and 0.5 m. Fig 4 shows the longitudinal temperature distribution beneath the roof of the belt-conveyor roadway under different mesh sizes.

**Fig 4:**
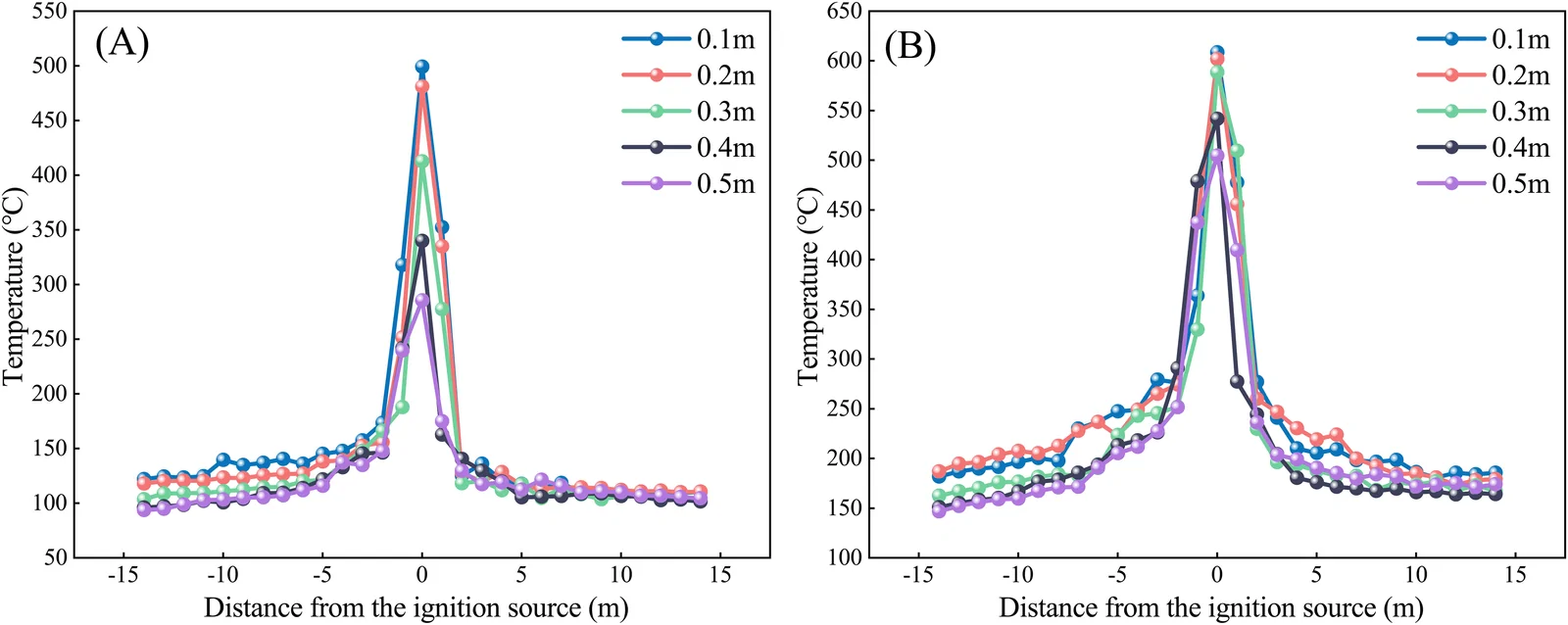
Comparison of ceiling temperatures obtained with different mesh sizes. (A) 2400 kW baseline thermal-source scenario. (B) 4800 kW thermal-source scenario.

As shown in Fig 4, decreasing the mesh size increased the calculated ceiling temperature near the source. Between the 0.1 and 0.2 m meshes, the peak-temperature differences were 3.61% and 1.14% for the 2400 and 4800 kW cases, respectively, while the corresponding mean absolute differences along the selected ceiling line were 5.05% and 4.52%. A mesh size of 0.2 m was therefore adopted, resulting in 1,237,500 computational cells.

### Experimental evaluation of the numerical model and construction of the full-scale U-shaped roadway model

To evaluate the capability of the numerical model to reproduce the principal smoke responses observed in the experiments, a corresponding numerical model was established based on the experimental geometry, ventilation conditions, and fire-source parameters. The temperature and CO concentration data measured at the upper monitoring point were selected for comparison with the experimental results. Because hot smoke generated by the fire mainly accumulates in the upper region of the roadway and spreads along the ventilation direction, the upper monitoring point is more sensitive to smoke propagation and thermal stratification. In addition, temperature and CO concentration represent the thermal and toxic hazards of the fire, respectively, and are therefore key parameters for evaluating roadway fire risk. The comparison curves between the experimental and simulation results are shown in Figs 5 and 6.

**Fig 5:**
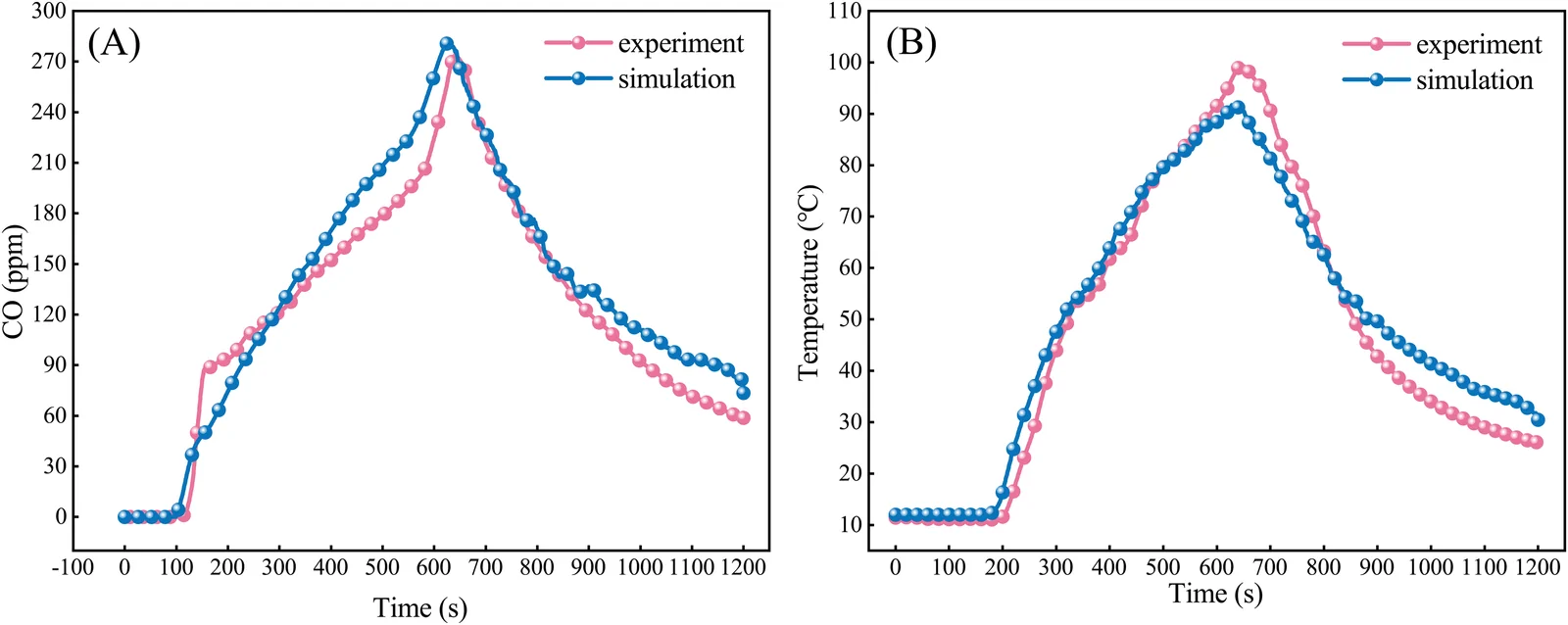
Comparison between experimental and simulation results under the 0.8 m/s condition. (A) Comparison of CO concentration curves. (B) Comparison of temperature curves.

**Fig 6:**
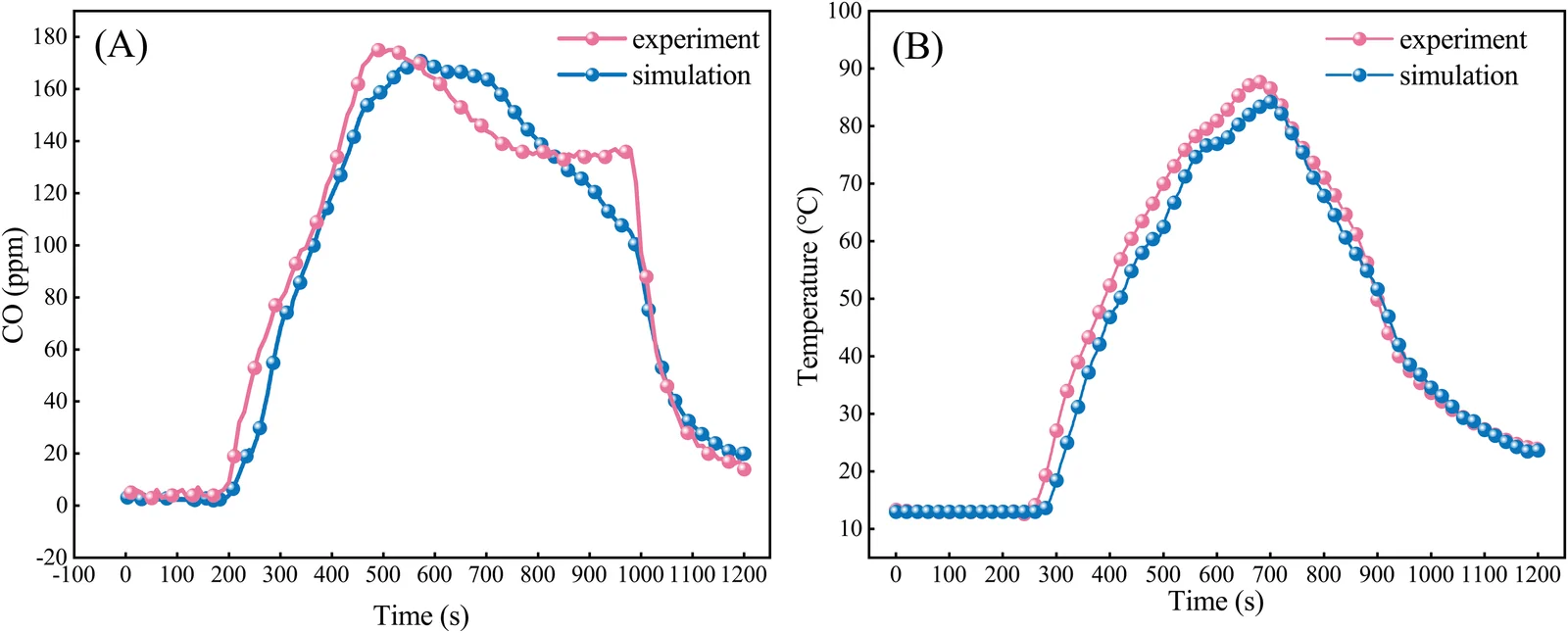
Comparison between experimental and simulation results under the 1.6 m/s condition. (A) Comparison of CO concentration curves. (B) Comparison of temperature curves.

As shown in Figs 5 and 6, under the two ventilation velocities of 0.8 m/s and 1.6 m/s, the simulated upper-layer CO concentration and temperature exhibited overall trends consistent with the experimental results. Both parameters showed limited variation during the initial stage, followed by a rapid increase, a peak, and a gradual decline. Under the 0.8 m/s condition, the simulated curves reproduced well the rising process and peak occurrence time of both CO concentration and temperature observed in the experiment. At 0.8 m/s, the deviations of the simulated CO and temperature peaks from the experimental values were 3.4% and −7.7%, respectively; at 1.6 m/s, the corresponding deviations were −2.3% and −3.9%. The maximum absolute peak deviation was 7.7%. Differences in local response and peak time may arise from experimental combustion fluctuations, sensor dynamics, the equivalent surface-source representation, and the prescribed species-source terms. The agreement in peak level and overall temporal evolution supports use of the model for comparative smoke-transport analysis under the specified conditions.

Based on the experimentally evaluated model, a full-scale three-dimensional numerical model of a U-shaped roadway was further constructed using PyroSim, with a belt-conveyor roadway in a coal mine taken as the engineering prototype, as shown in Fig 7. The model consisted of a belt-conveyor roadway, a connecting roadway, a working face and a return-air roadway. The belt-conveyor roadway and return-air roadway were both 120 m long, 6 m wide and 5 m high, while the connecting roadway and working face were each 60 m long. All roadways were arranged horizontally with an inclination angle of 0°, and the cross-section adopted a three-centered arch with straight sidewalls. To capture the key parameter variations during smoke spread, temperature and CO volume-fraction sensors were arranged in each roadway. The monitoring height was set at 4.0 m above the floor to characterize hot-smoke accumulation in the upper region of the roadway. Meanwhile, temperature and visibility were selected as the principal slice-output variables for analyzing the spatial distributions of the thermal field and visibility. Taking the fire-source centre as the longitudinal origin of the belt-conveyor roadway, negative and positive coordinates denote upstream and downstream positions, respectively. Monitoring points in the belt-conveyor roadway were located at −50, −40, −30, −15, 0, 15, 30, and 45 m, and working-face monitoring points were located 0, 15, 30, 45, and 60 m from its entrance. All sensors were positioned 4.0 m above the floor. The fire-source centre was 70 m from the belt-conveyor-roadway inlet.

**Fig 7:**
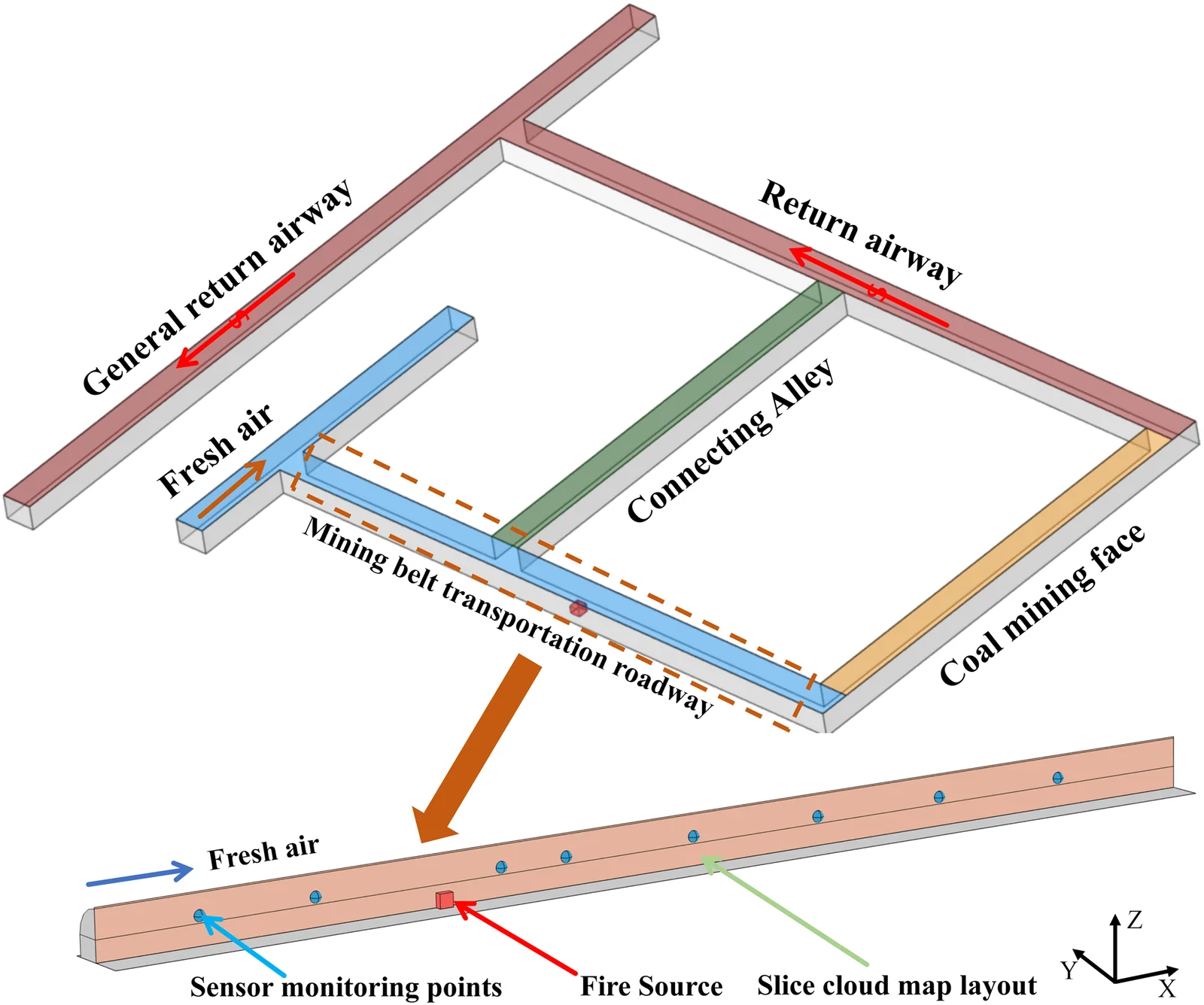
Schematic diagram of the simplified mine roadway model and monitoring-point arrangement.

When establishing the numerical model of the U-shaped roadway conveyor belt fire, several simplifications were introduced to highlight the smoke-spread behavior within the roadway network while ensuring computational efficiency and model stability [42,43]. The corresponding boundary conditions are listed in Table 3.

**Table 3:** Boundary conditions for the numerical model of the U-shaped belt-conveyor roadway fire.

Boundary Type	Model Domain	Parameter Settings
Inlet Boundary	Belt-conveyor roadway inlet	Velocity inlet, wind speeds set to 0.8, 1.2, 1.6, 2.0, 2.4, 2.8, 3.2 m/s; temperature 285.15 K
Outlet Boundary	Return-air roadway outlet	Pressure outlet, ambient pressure 101,325 Pa
Wall Boundary	Roadway walls	No-slip adiabatic boundary; thermal conduction to the surrounding rock neglected
Fire-source Boundary	Fire surface	Burning surface; prescribed thermal-source intensity: 2400 / 4800 kW
Smoke Boundary	Fire region	Experimentally constrained CO and CO_2_ release functions; equivalent-combustion parameters: CO yield 0.032, soot yield 0.060, radiative fraction 0.35; identical settings applied to both thermal-source levels
Mesh Parameters	Full domain	Structured mesh, 0.2 m × 0.2 m × 0.2 m, total 1,237,500 cells
Simulation Duration	Time domain	600 s, time step 0.1 s
Turbulence Model	Full domain	Large-eddy simulation, Smagorinsky subgrid-scale model
Combustion Model	Fire region	Mixture-fraction combustion model
Initial Conditions	Full domain	Temperature 285.15 K, pressure 101,325 Pa, wind velocity 0 m/s

(1) The fire was represented by a localized equivalent surface source with prescribed heat release rate and species-release parameters.
(2) The effective source area was fixed at 1.0 m^2^. The 2400 kW case was used as the baseline thermal-source scenario, and the 4800 kW case represented a twofold thermal-source condition; continuous flame spread along the belt was not included in the equivalent-source formulation.
(3) The combustion process was described using a one-step mixing-controlled combustion model. The combustible volatiles generated by conveyor-belt pyrolysis were simplified as an equivalent fuel, and the combustion reaction was represented based on the mixture-fraction approach.
(4) The experimentally constrained CO and CO_2_ release functions and the prescribed soot parameters were applied consistently to both thermal-source levels, providing a common species-source condition for the comparative analysis.
(5) The belt was stationary. Material melting, dripping, charring, flame spread, interactions between upper and lower belts, and HCl release were outside the equivalent source formulation.
(6) Roadway ventilation was simplified as a steady longitudinal flow. The inlet velocity was kept constant within each case, and the effects of airflow fluctuation, ventilation-facility adjustment and local unsteady disturbances on the flow field were neglected.
(7) The roadway cross-section was constructed as a regular three-centered arch with straight sidewalls. Fine-scale disturbances induced by local components such as idlers and supports were neglected, and the roadway walls were treated as no-slip adiabatic boundaries.

### Simulation results analysis and discussion

Figs 8 and 9 present the temperature and visibility distributions in the belt-conveyor main roadway under the 2400 kW baseline and 4800 kW thermal-source scenarios, respectively. As shown in Figs 8(A) and 9(A), the temperature field within the fire-source roadway is primarily governed by the combined effects of the buoyant plume and the longitudinal ventilation momentum. At a ventilation velocity of 0.8 m/s, the longitudinal airflow is relatively weak, allowing the hot smoke above the fire to rise rapidly under buoyancy and spread along the roof. This forms a relatively continuous upper hot-smoke layer, with a large high-temperature region near the fire source. At 1.2 m/s, the deflection effect of the longitudinal airflow on the fire plume is enhanced, causing the high-temperature core region to tilt toward the downstream side, although a noticeable temperature rise persists near the roof, indicating that this wind speed is still insufficient to fully suppress buoyancy-driven smoke accumulation. At 1.6 m/s, the plume deflection becomes more pronounced, the upstream high-temperature region further contracts, and smoke transport gradually transitions from buoyancy-dominated to airflow-inertia-controlled. Previous studies have shown that smoke back-layering under longitudinal ventilation is controlled by a critical velocity; when the wind speed is below the critical value, fire-source buoyancy can induce the hot smoke along the roof to extend upstream, whereas near or above the critical velocity, the back-layering length is significantly reduced [43–45]. In Figs 8(A) and 9(A), the upstream high-temperature region is clearly weakened under the 2.0 m/s condition, indicating strong suppression of near-fire buoyant back-layering at this ventilation velocity. As the wind speed increases further to 2.4, 2.8 and 3.2 m/s, the high-temperature zone shifts mainly to the downstream side of the fire, the ceiling temperature decays more rapidly along the flow direction, and the high-temperature core near the fire is compressed by the forced airflow, showing a temperature distribution increasingly dominated by mechanical ventilation.

**Fig 8:**
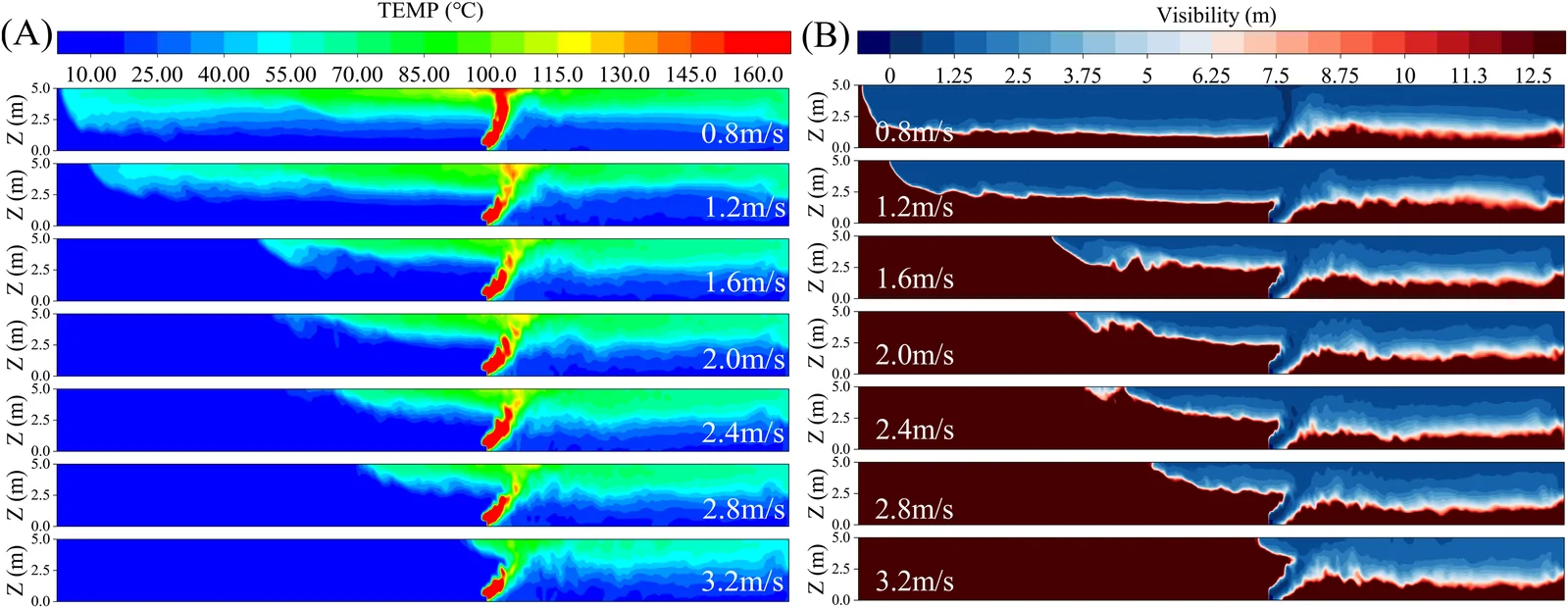
Slice contours of the belt-conveyor main roadway under the 2400 kW baseline thermal-source scenario. (A) Temperature distribution. (B) Visibility distribution.

**Fig 9:**
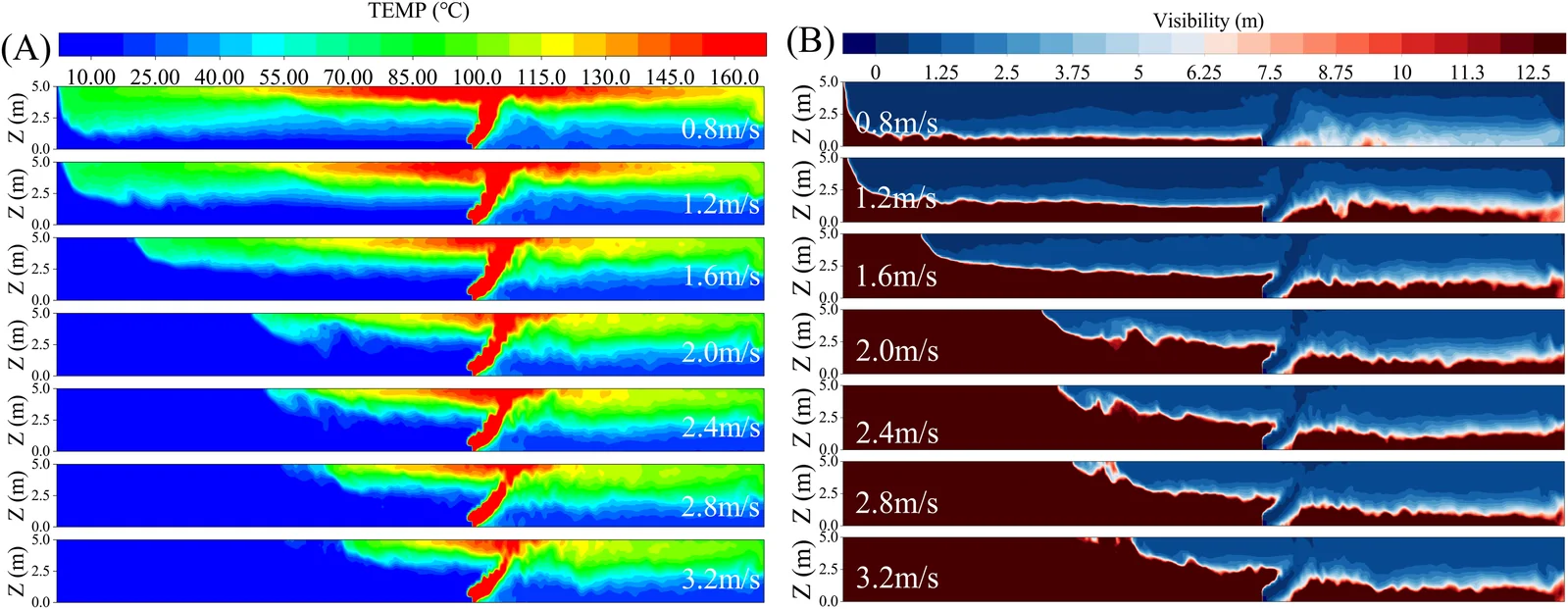
Slice contours of the belt-conveyor main roadway under the 4800 kW thermal-source scenario. (A) Temperature distribution. (B) Visibility distribution.

Comparing Figs 8(A) and 9(A), it is evident that the high-temperature region under the 4800 kW condition is significantly larger than that under 2400 kW. The buoyant plume above the fire is stronger, and the medium-to-high-temperature smoke layer near the roof is more continuous. Particularly under the low ventilation velocities of 0.8 and 1.2 m/s, the higher prescribed thermal-source intensity generates a stronger buoyant plume, resulting in more pronounced accumulation of hot smoke near the roof. At 2.0 m/s, the 4800 kW case still exhibits a relatively large medium-to-high-temperature region downstream of the source, reflecting the stronger thermal response associated with the higher source level. Figs 8(B) and 9(B) show that, at low ventilation velocities, the low-visibility region is concentrated near the fire source and within the upper smoke layer. As ventilation velocity increases, the region of reduced visibility extends downstream. At 2.4 m/s and above, the peak temperature and near-source high-temperature region decrease, whereas smoke transport by the longitudinal airflow increases. Thus, stronger ventilation suppresses near-source thermal accumulation but can enlarge the downstream low-visibility region, consistent with previous roadway-fire studies [18].

Figs 10 and 11 show the temperature and visibility distributions in the working-face roadway under the 2400 kW baseline and 4800 kW thermal-source scenario, respectively. As observed in Figs 10(A) and 11(A), the working-face roadway is not directly affected by the fire source; its temperature field is primarily controlled by hot smoke transported from the belt-conveyor main roadway, resulting in overall lower temperatures compared to the fire-source roadway. At a ventilation velocity of 0.8 m/s, the hot smoke entering the working face is mainly concentrated in the upper part of the roadway, with pronounced vertical thermal stratification, indicating that buoyancy-driven layering persists in the far-field region. Under 1.2 m/s and 1.6 m/s conditions, the upper temperature rise region extends along the roadway, demonstrating that increased ventilation enhances the transport capacity of hot smoke into the working face. At 2.0 m/s, the temperature distribution becomes more uniform and the thickness of the upper hot layer decreases, suggesting that shear mixing between the longitudinal airflow and the hot smoke layer is strengthened, reducing the stability of the smoke layer. Previous studies have shown that increasing longitudinal ventilation velocity significantly affects the stable stratification length and interface position of smoke layers, and strong shear can disrupt the thermal smoke layer, promoting mixing and diffusion into lower regions [24,25]. When the wind speed further increases to 2.4, 2.8, and 3.2 m/s, the overall temperature rise in the working-face roadway decreases, and the vertical stratification becomes less pronounced, indicating that the far-field thermal hazard gradually transitions from upper-layer stratified accumulation to mixed transport controlled by ventilation dilution.

**Fig 10:**
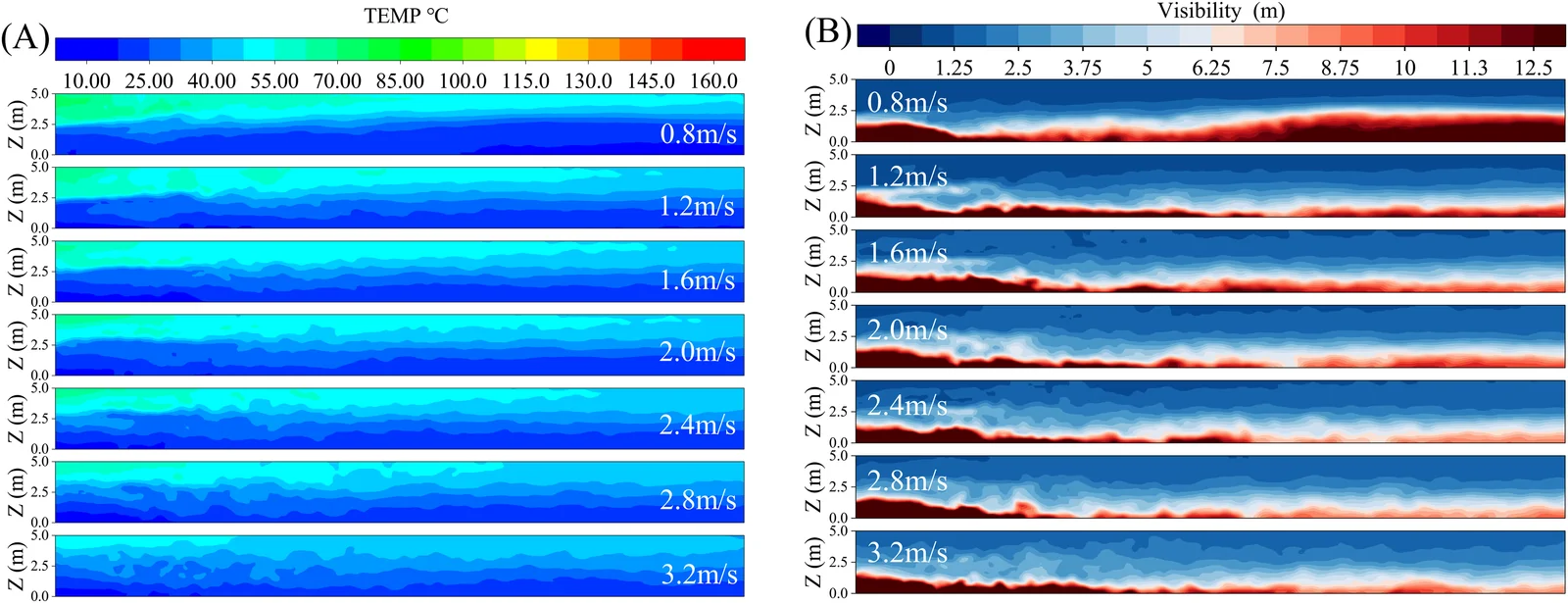
Slice contours of the working-face roadway under the 2400 kW baseline thermal-source scenario. (A) Temperature distribution. (B) Visibility distribution.

**Fig 11:**
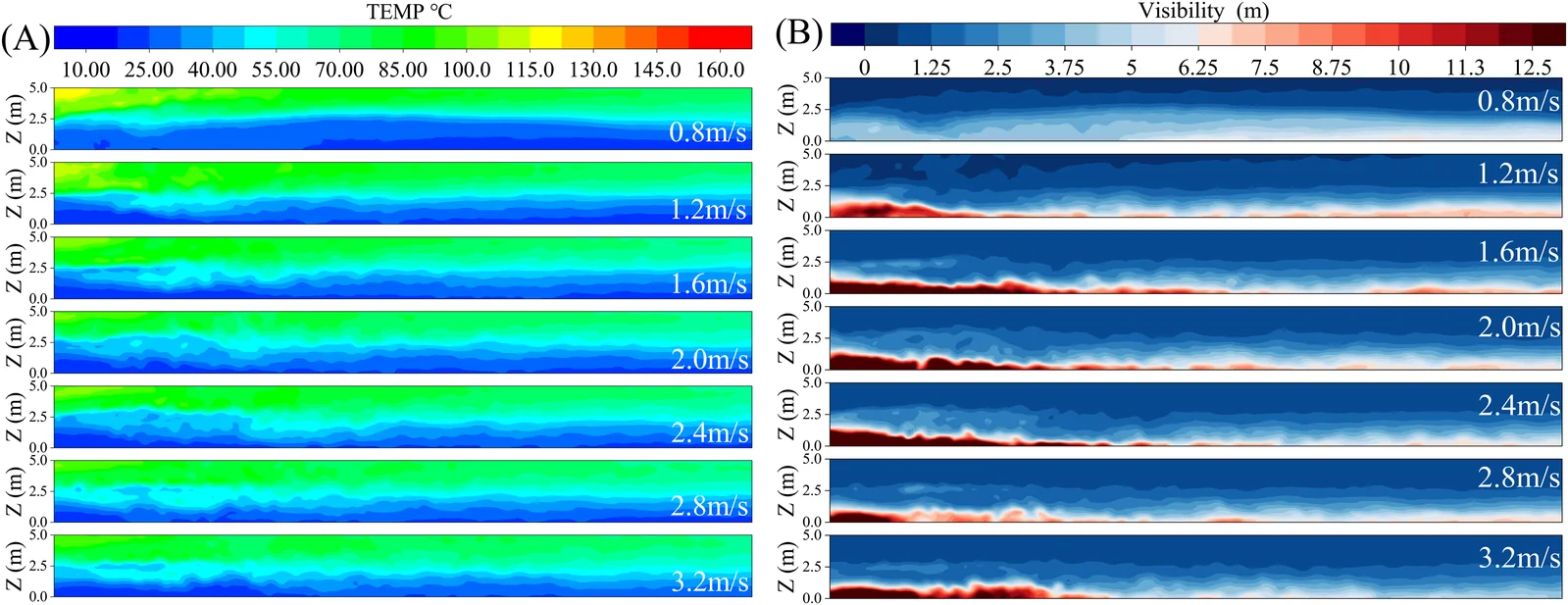
Slice contours of the working-face roadway under the 4800 kW thermal-source scenario. (A) Temperature distribution. (B) Visibility distribution.

Comparing Figs 10(A) and 11(A), the 4800 kW thermal-source scenario produces a larger upper temperature-rise region and a more continuous medium-temperature zone in the working face, indicating a stronger thermal response than that under the 2400 kW baseline condition. Figs 10(B) and 11(B) further show that the low-visibility region does not contract in direct proportion to the temperature decrease. At 0.8 and 1.2 m/s, smoke remains concentrated in the upper space under buoyancy-driven stratification. At 1.6 and 2.0 m/s, stronger longitudinal flow increases lateral and vertical smoke diffusion, and the lower roadway begins to be affected. At 2.4 m/s and above, the thermal hazard decreases, but smoke continues to move along the working face. Temperature therefore reflects heat transport and attenuation, whereas visibility is additionally governed by soot transport, turbulent diffusion, and local entrainment; both variables are required to characterize the working-face hazard.

Figs 12 and 13 show the temperature and visibility distributions in the connecting roadway under the 2400 kW baseline and 4800 kW thermal-source scenario, respectively. As shown in Figs 12(A) and 13(A), the temperature in the connecting roadway is significantly lower than in the belt-conveyor main roadway and the working-face roadway, indicating that this region is mainly influenced by far-field smoke intrusion and low-temperature smoke diffusion. At 0.8 m/s, a noticeable temperature rise is still observed in the upper part of the connecting roadway, with hot smoke distributed near the roof and clear vertical stratification, suggesting that buoyancy remains an important driving force for smoke entry under low ventilation conditions. Under 1.2 m/s and 1.6 m/s, the upper temperature rise region expands, showing that smoke from the main roadway is increasingly transported into the connecting roadway by ventilation. At 2.0 m/s, the temperature field under the 2400 kW scenario decreases markedly, whereas the 4800 kW case retains a more evident upper-layer temperature rise, reflecting the stronger far-field thermal response associated with the higher prescribed source level. When the ventilation velocity increases to 2.4 m/s and above, the overall temperature in the connecting roadway declines and the high-temperature zone nearly disappears, indicating that forced ventilation enhances cold-air supply and convective heat transfer, reducing the accumulation of hot smoke in the connecting roadway.

**Fig 12:**
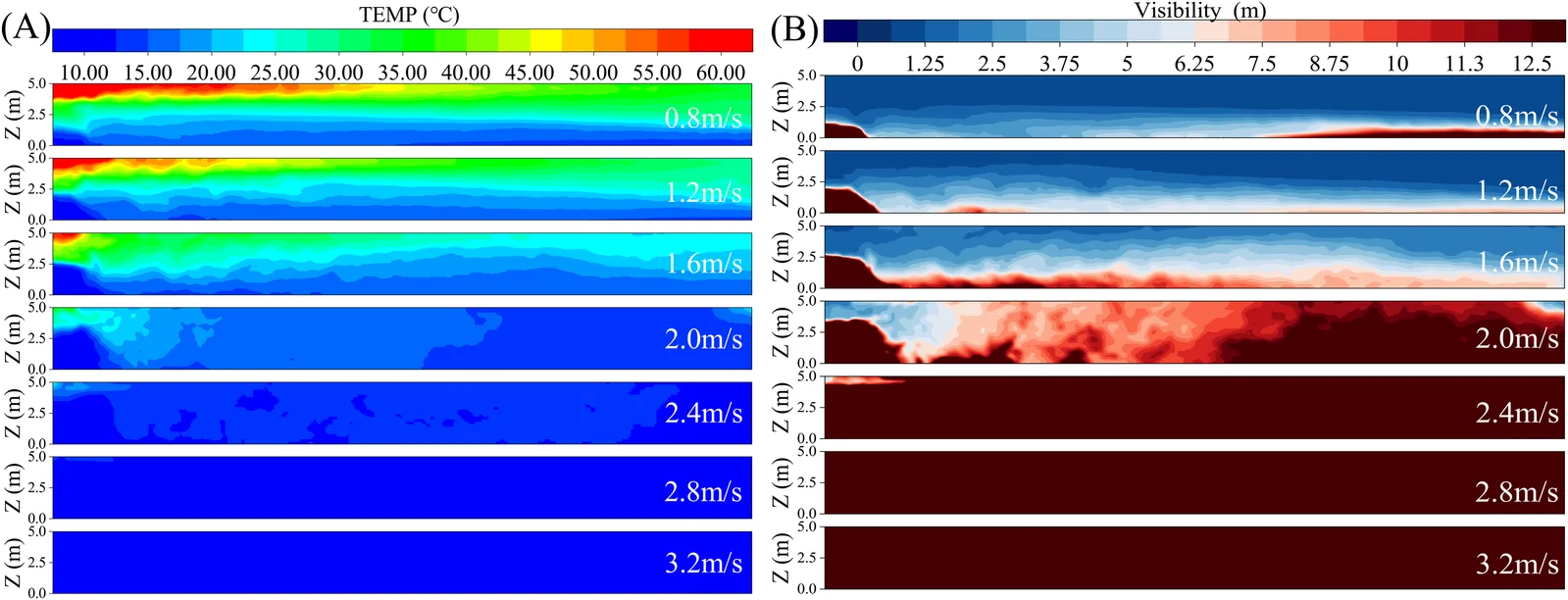
Slice contours of the connecting roadway under the 2400 kW baseline thermal-source scenario. (A) Temperature distribution. (B) Visibility distribution.

**Fig 13:**
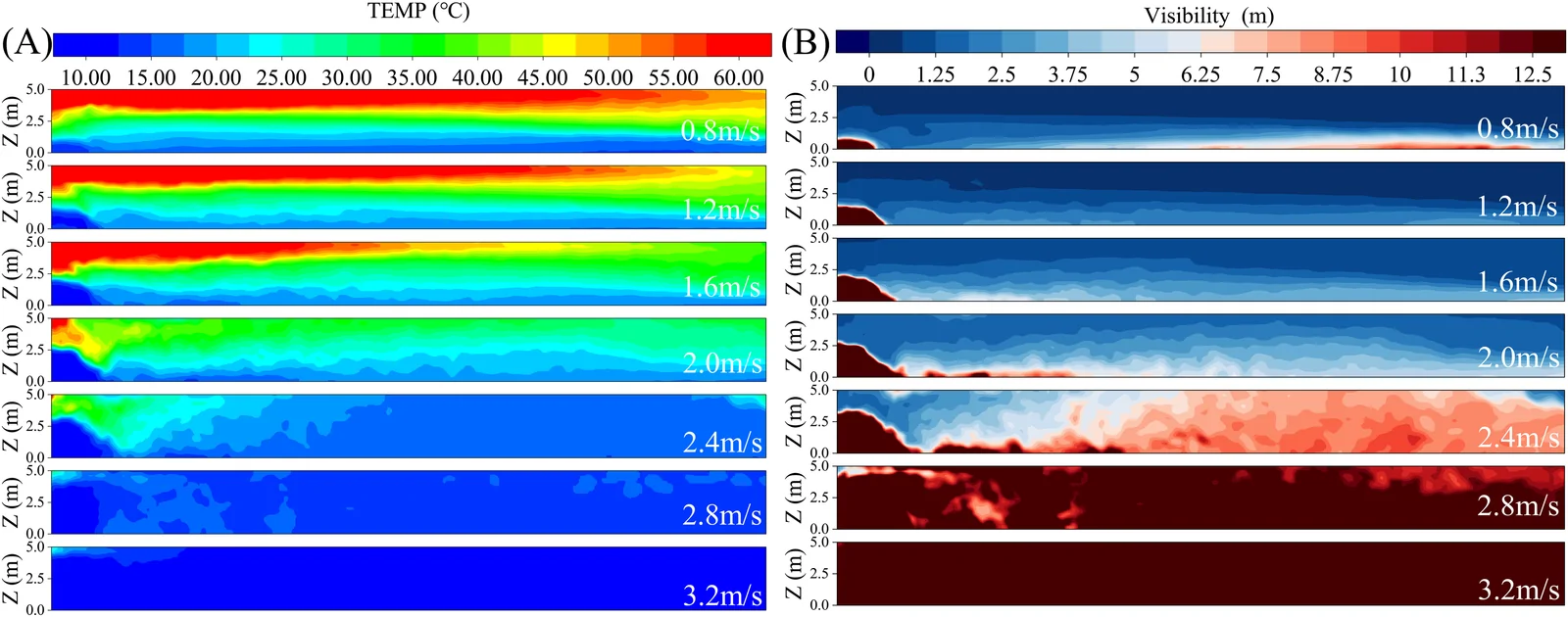
Slice contours of the connecting roadway under the 4800 kW thermal-source scenario. (A) Temperature distribution. (B) Visibility distribution.

Figs 12(B) and 13(B) show that the visibility distribution does not fully coincide with the temperature field. At 0.8 m/s, smoke mainly enters the upper part of the connecting roadway, and the low-visibility region is limited. At 1.2 and 1.6 m/s, increasing longitudinal momentum strengthens smoke transport into the connecting roadway and enlarges the local low-visibility region. Near 2.0 m/s, the temperature field decreases while the region of reduced visibility continues to expand, indicating a transition from buoyancy-driven intrusion to ventilation-entrained and turbulence-mixed transport. At 2.4 m/s and above, the thermal hazard diminishes further, but cooler smoke-laden flow can occupy a larger portion of the connecting-roadway cross-section. In complex roadway networks, the connecting roadway lies in a main-flow turning and bifurcation zone, where local pressure differences, backflow vortices and shear entrainment jointly modify smoke transport paths. Previous studies have indicated that smoke back-layering and distribution in connecting roadways differ from straight roadways; fire-source location, node spacing, and ventilation pressure gradients all affect the critical conditions for smoke entry into branch roadways and the diffusion range [13,23]. Therefore, the fire risk in the connecting roadway is not only reflected by temperature rise but also by widespread low-temperature smoke, which degrades visibility and increases difficulty for personnel evacuation.

Comparing the slice results from the belt-conveyor main roadway, the working-face roadway, and the connecting roadway shows that smoke propagation within the U-shaped roadway varies with ventilation velocity and prescribed thermal-source intensity, while the turning and connecting regions further modify the local flow and smoke-transport patterns. Under low wind speeds, buoyancy effects dominate, and high-temperature smoke tends to accumulate near the roof, forming a relatively stable stratified layer. The thermal hazard is most pronounced in the belt-conveyor main roadway. As the wind speed increases to the 1.6–2.0 m/s range, the smoke-control mechanism enters a transitional stage: back-layering and near-source heat accumulation are suppressed, while the smoke layer stability decreases and transport into the working-face and connecting roadways becomes more significant. When wind speeds exceed 2.4 m/s, the near-source high-temperature zone is further compressed, the temperature hazard decreases overall, but smoke disperses more prominently into the far-field roadways under ventilation. Increasing the prescribed thermal-source intensity from 2400 to 4800 kW strengthens plume buoyancy and modifies the smoke-transport field, producing a larger high-temperature region in the belt-conveyor roadway and a greater temperature rise in the working face. Because the same species-source formulation was applied at both thermal-source levels, the corresponding CO and visibility fields reflect the redistribution, dilution, and mixing of the prescribed smoke source within the modified flow field. This indicates that ventilation control in belt-conveyor roadway fires cannot focus solely on increasing airflow or reducing near-source temperatures. Instead, a balance must be achieved among suppressing upstream smoke back-layering, controlling near-source high-temperature accumulation, and preventing rapid smoke spread into the working-face and connecting roadways. Within the U-shaped configuration, smoke underwent successive turning and redistribution along the belt-conveyor roadway–working face–connecting roadway–return-air roadway path. At the junctions and bends, momentum redistribution favoured local low-velocity and recirculating flow near the inner corners, while shear and entrainment were enhanced along the outer sides [10,23].These flow characteristics are consistent with the smoke bifurcation, local accumulation, deformation of the upper smoke layer, and renewed downstream transport observed after turning. Accordingly, the present analysis characterizes the geometry-associated redistribution of smoke along the belt-conveyor roadway–working face–connecting roadway–return-air roadway path. Quantitative isolation of the geometric contribution would require a corresponding reference configuration under otherwise identical source and ventilation conditions.

The 4800 kW thermal-source scenario produced a stronger thermal response than the 2400 kW baseline scenario, while the associated smoke and visibility fields showed corresponding changes in transport and spatial redistribution. Temperature and CO histories at representative points in the belt-conveyor roadway and working face were therefore analysed to compare response onset, peak level, duration, and spatial attenuation under different ventilation velocities. Peak values were subsequently nondimensionalized to characterize changes in smoke-transport behaviour with ventilation velocity and to identify the corresponding transition range under the specified source and roadway conditions.

To further quantify hot-smoke transport along the principal flow path under the 4800 kW thermal-source scenario, temperature and CO data from the 4.0 m monitoring points in the belt-conveyor roadway and working face were analyzed. This approach accounts for the fact that smoke in the connecting roadway is influenced by backflow and local deflection from the main plume, making its measurements partially indirect. The analysis thus emphasizes the primary smoke transport and thermal hazard within the main airflow path.

Under the 0.8 m/s ventilation condition, the temperature and CO concentration at representative monitoring points exhibited pronounced low-velocity accumulation characteristics. As shown in Fig 14, the 0 m monitoring point in the belt roadway responded first and showed the most rapid increase. At 600 s, the temperature and CO concentration reached approximately 200 °C and 570 ppm, respectively, substantially higher than those at the other monitoring points. The simultaneous temperature and CO responses indicate that the near-source monitoring location was the principal region of thermal and toxic-gas exposure under low-velocity ventilation. Notable temperature rise and CO accumulation were also observed at the upstream monitoring points of −50 m, −40 m, and −30 m, suggesting that the airflow momentum under low-velocity ventilation was insufficient to overcome the buoyancy effect induced by the fire source. As a result, high-temperature smoke carrying CO propagated upstream along the upper part of the roadway, leading to localized enrichment on the upstream side. The responses at the working-face monitoring points generally lagged behind those in the belt roadway. At the 0 m point of the working face, the temperature and CO concentration were approximately 120 °C and 465 ppm, respectively, and both decreased gradually along the working-face direction. This indicates that, after entering the far-field region, smoke was progressively attenuated by flow deflection, cooler-air entrainment, turbulent mixing, and dilution, resulting in a gradual reduction in thermal intensity and toxic-gas concentration. Together with the sustained responses at the upstream monitoring points, these results indicate that buoyancy dominated smoke transport at 0.8 m/s, producing roof-level back-layering and continued smoke intrusion into the working face.

**Fig 14:**
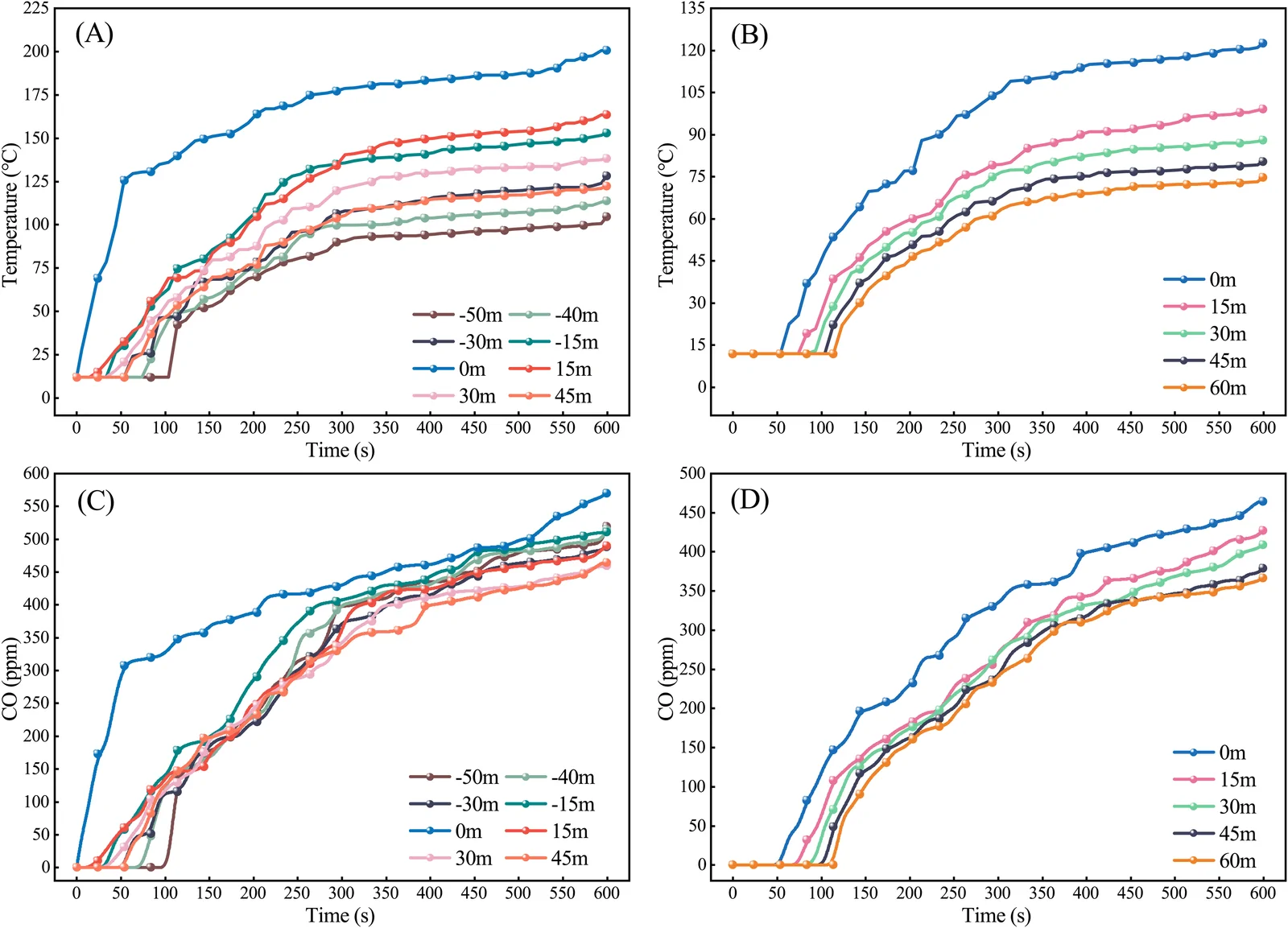
Temperature and CO concentration at representative points under 0.8 m/s ventilation. (A) Temperature in the belt roadway. (B) Temperature at the working face. (C) CO concentration in the belt roadway. (D) CO concentration at the working face.

When the roadway air velocity increased to 1.2 m/s, the peak temperature and CO concentration at representative monitoring points decreased compared with those under the 0.8 m/s condition, indicating that the regulatory effect of roadway airflow on smoke transport began to intensify within the low-velocity range. As shown in Fig 15, the 0 m monitoring point in the belt roadway still exhibited the strongest response. At 600 s, the temperature was approximately 180 °C and the CO concentration was approximately 500 ppm, suggesting that the vicinity of the fire source remained the primary region for heat and toxic gas accumulation.

**Fig 15:**
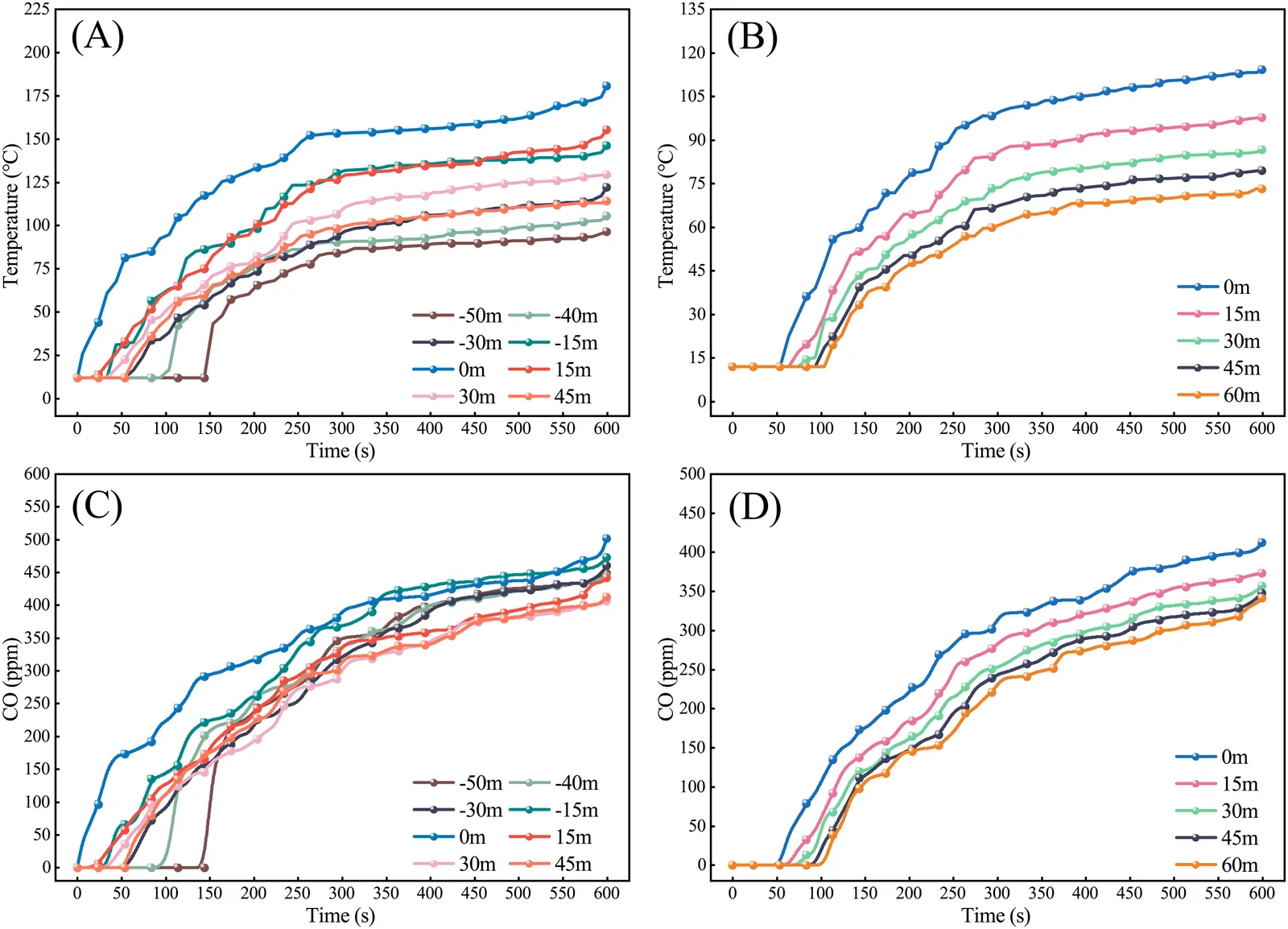
Temperature and CO concentration at representative points under 1.2 m/s ventilation. (A) Temperature in the belt roadway. (B) Temperature at the working face. (C) CO concentration in the belt roadway. (D) CO concentration at the working face.

Compared with the 0.8 m/s condition, the temperature rise and CO increase at the upstream monitoring points of −50 m, −40 m, and −30 m were reduced, indicating that both the extent of smoke backflow and the intensity of accumulation were partially weakened. However, the buoyancy effect was still not fully suppressed. At the 0 m monitoring point of the working face, the temperature and CO concentration at 600 s were approximately 115 °C and 410 ppm, respectively, remaining higher than those at other locations and decreasing progressively along the working-face direction. Compared with the 0.8 m/s case, the weaker upstream response and the persistent response at the working face indicate that ventilation at 1.2 m/s partially suppressed smoke back-layering but did not prevent smoke intrusion along the downstream path.

Under the 1.6 m/s ventilation condition, smoke transport began to shift from a buoyancy-dominated regime to one jointly controlled by buoyancy and roadway airflow. As shown in Fig 16, the 0 m monitoring point in the belt roadway remained the location with the strongest response. At 600 s, the temperature and CO concentration were approximately 168 °C and 438 ppm, respectively, both lower than those under the 1.2 m/s condition. The lower near-source temperature and CO responses than those at 1.2 m/s reflect stronger convective heat removal and smoke dilution at the monitoring section. The responses at the upstream monitoring points, such as −50 m and −40 m, were markedly delayed, with only limited increases in temperature and CO concentration. This suggests that smoke backflow was more strongly suppressed and that the upstream smoke-enrichment region was substantially shortened. Meanwhile, the downstream monitoring points at 15 m, 30 m, and 45 m maintained relatively high levels, indicating that smoke transport became increasingly concentrated downstream and that the directional propagation of the main smoke plume was strengthened. At the 0 m monitoring point of the working face, the temperature and CO concentration at 600 s were approximately 107 °C and 352 ppm, respectively, and both decreased gradually along the working-face direction. The simultaneous attenuation of the upstream response and persistence of the working-face response indicate that, at 1.6 m/s, smoke transport began to shift from buoyancy-supported back-layering toward ventilation-directed downstream advection.

**Fig 16:**
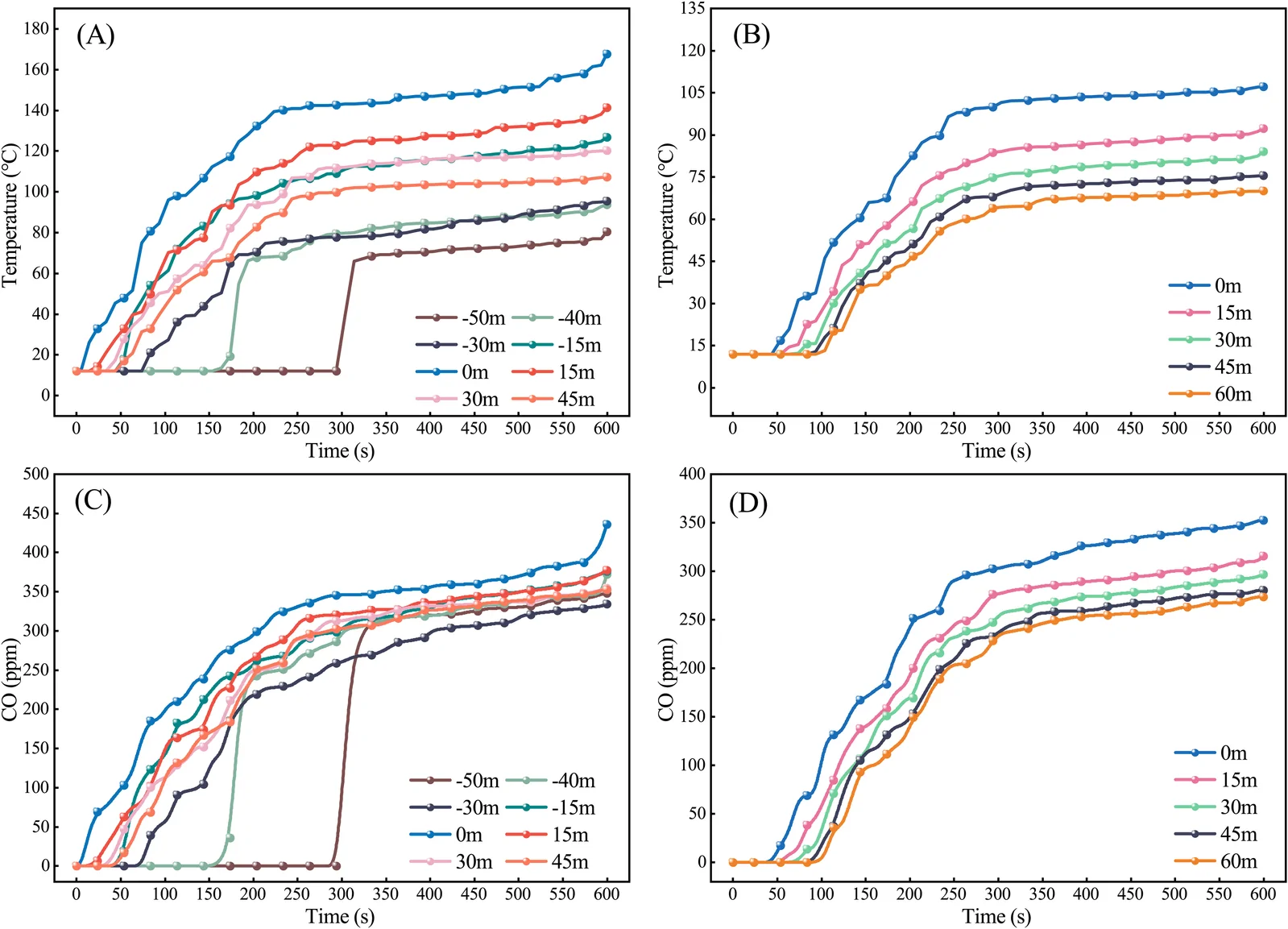
Temperature and CO concentration at representative points under 1.6 m/s ventilation. (A) Temperature in the belt roadway. (B) Temperature at the working face. (C) CO concentration in the belt roadway. (D) CO concentration at the working face.

Under the 2.0 m/s ventilation condition, the responses at the monitoring points upstream of the fire source were further weakened, indicating that smoke backflow in the belt roadway was strongly suppressed. As shown in Fig 17, at the 0 m monitoring point, the temperature and CO concentration at 600 s were approximately 155 °C and 365 ppm, respectively, both lower than those under the 1.6 m/s condition. The reduced source-point response and the near-baseline values at the remote upstream points show that longitudinal airflow increasingly restricted long-distance smoke back-layering at 2.0 m/s. At the −40 m monitoring point, both temperature and CO concentration remained close to their initial levels. Although a certain temperature rise was observed at the −50 m point during the later stage, the CO concentration remained relatively low, indicating that the upstream accumulation range of toxic gases was markedly reduced. The downstream monitoring points at 15 m, 30 m, and 45 m still exhibited relatively strong responses, suggesting that smoke was mainly transported along the direction of roadway airflow and that the directionality of the main smoke plume became more pronounced. At the 0 m monitoring point of the working face, the temperature and CO concentration were approximately 103 °C and 325 ppm, respectively, indicating that a certain degree of accumulation still persisted. Nevertheless, the working-face response remained evident, demonstrating that the suppression of upstream back-layering was accompanied by continued smoke transport toward the downstream roadway and working face.

**Fig 17:**
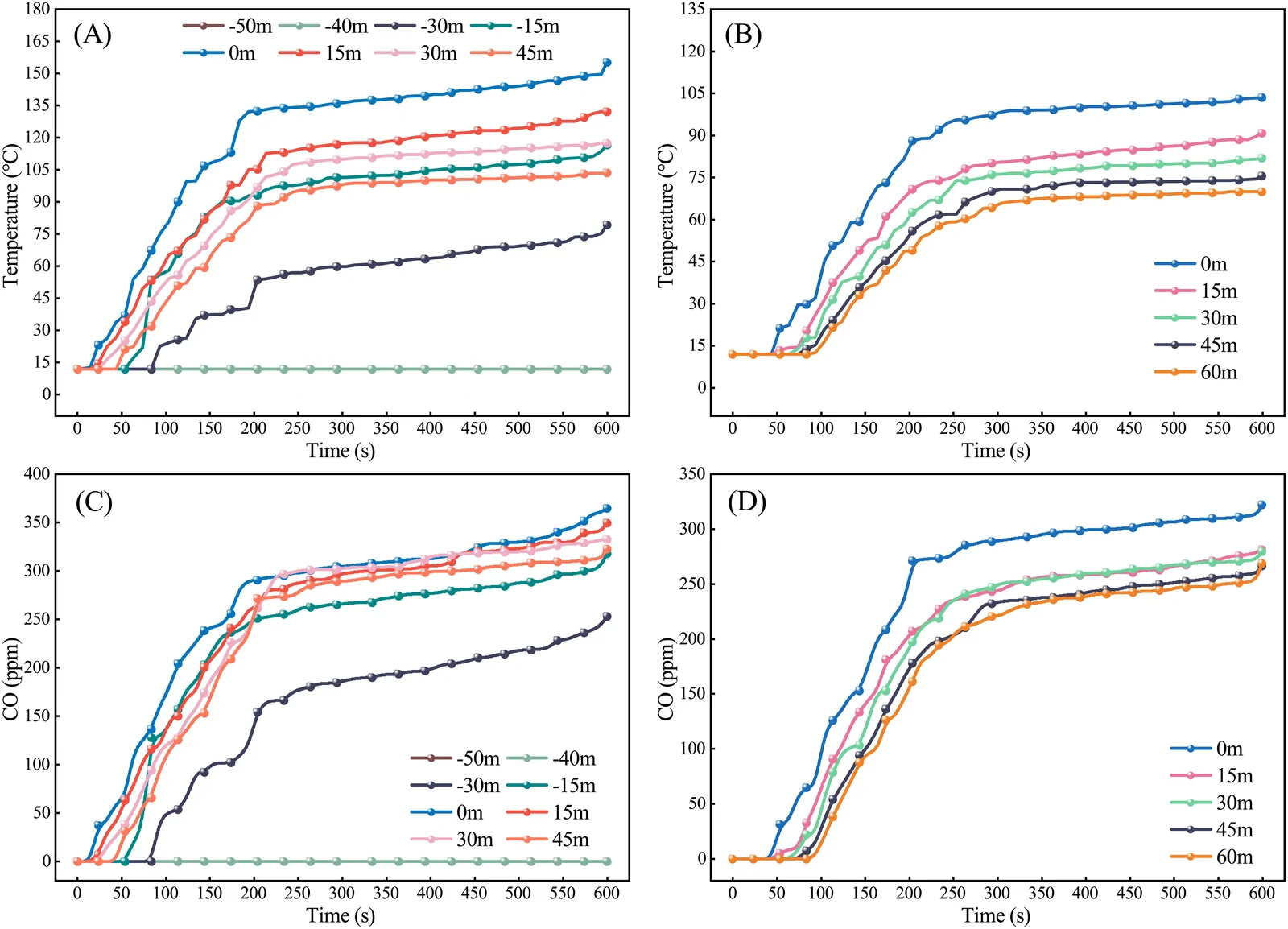
Temperature and CO concentration at representative points under 2.0 m/s ventilation. (A) Temperature in the belt roadway. (B) Temperature at the working face. (C) CO concentration in the belt roadway. (D) CO concentration at the working face.

When the roadway air velocity increased to 2.4 m/s, upstream smoke backflow was further restricted, and the temperature and CO responses in the region upstream of the fire source were significantly weakened. As shown in Fig 18, the temperature and CO concentration at the −50 m and −40 m monitoring points in the belt roadway remained close to their initial levels, while only a weak response was observed at the −30 m point during the later stage. Remote upstream back-layering was largely suppressed at 2.4 m/s, with near-baseline responses at −50 and −40 m and only a weak late response at −30 m. However, the 0 m monitoring point remained the location with the strongest near-source response, reaching approximately 153°C and 378 ppm at 600 s. The downstream monitoring points at 15 m, 30 m, and 45 m maintained relatively high levels, indicating that smoke was mainly transported along the direction of roadway airflow. In the working-face region, the 0 m monitoring point recorded a temperature of approximately 105 °C and a CO concentration of approximately 333 ppm at 600 s. The differences among monitoring points along the working face became smaller, reflecting enhanced far-field smoke mixing under higher air velocity. The smaller differences among the working-face monitoring points reflect stronger smoke mixing; however, the sustained CO response indicates that increased ventilation redistributed the toxic-gas hazard downstream rather than eliminating it.

**Fig 18:**
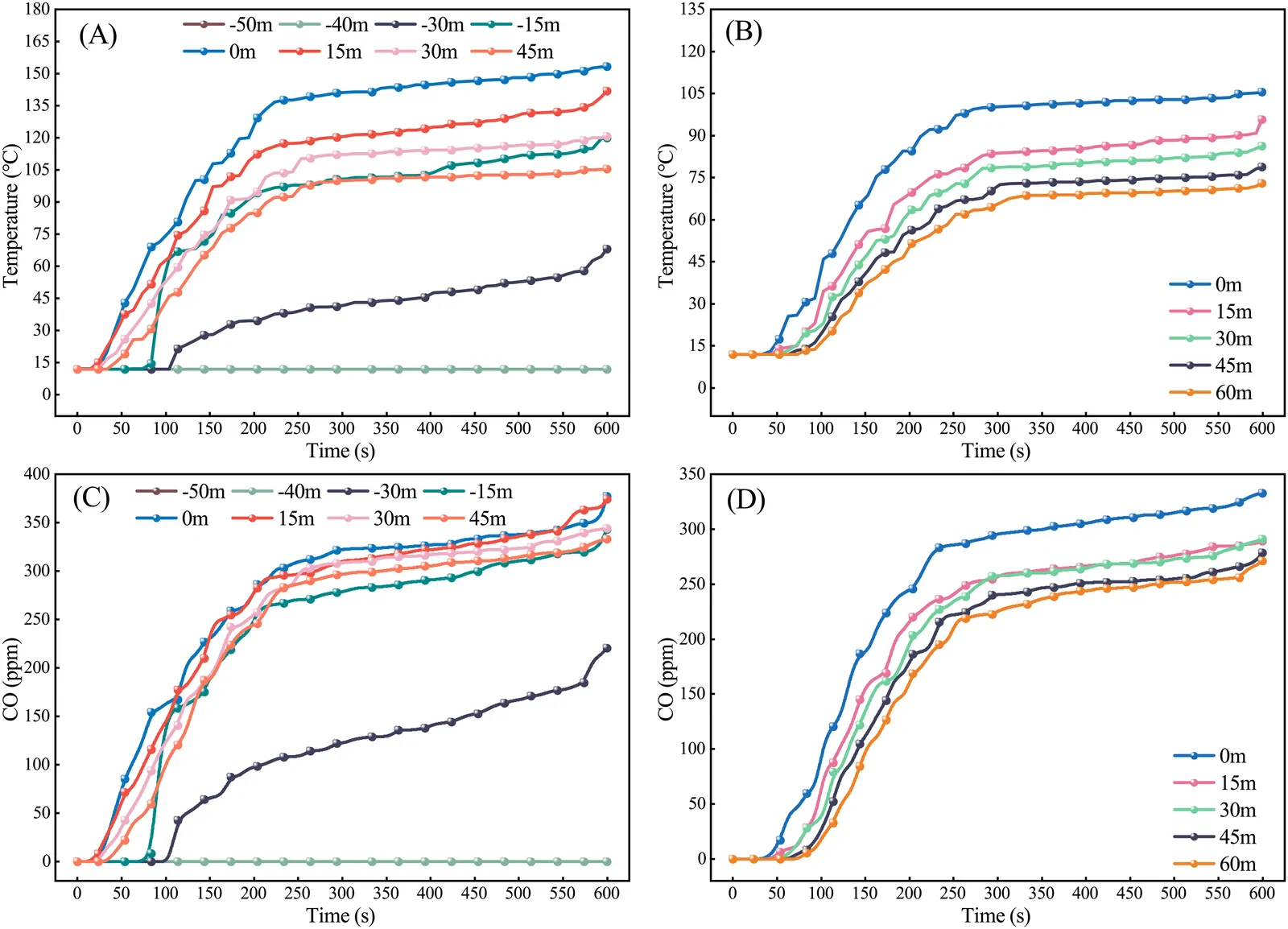
Temperature and CO concentration at representative points under 2.4 m/s ventilation. (A) Temperature in the belt roadway. (B) Temperature at the working face. (C) CO concentration in the belt roadway. (D) CO concentration at the working face.

Under the 2.8 m/s ventilation condition, the stronger roadway airflow further compressed the upstream smoke-affected region, resulting in a more stable smoke-transport direction in the belt roadway. As shown in Fig 19, the temperature and CO concentration at the −50 m and −40 m monitoring points remained close to their initial levels, while only a limited response was observed at the −30 m point. This indicates that the upstream enrichment of hot smoke and CO was substantially weakened. Near the fire source, the 0 m monitoring point recorded a temperature of approximately 158 °C and a CO concentration of approximately 390 ppm at 600 s. The downstream monitoring points at 15 m, 30 m, and 45 m still maintained relatively high levels, indicating that the main smoke plume was continuously transported downstream under the action of stronger roadway airflow. In the working-face region, the temperature and CO concentration at the 0 m monitoring point were approximately 104 °C and 330 ppm, respectively, while the differences among the remaining monitoring points were further reduced. This reflects enhanced smoke mixing and diffusion in the far-field region. Compared with the near-source backflow, which was largely controlled, the CO concentration at the working face remained relatively high, suggesting that high air velocity did not completely reduce the far-field toxic-gas risk but instead promoted continuous smoke transport along the main ventilation path.

**Fig 19:**
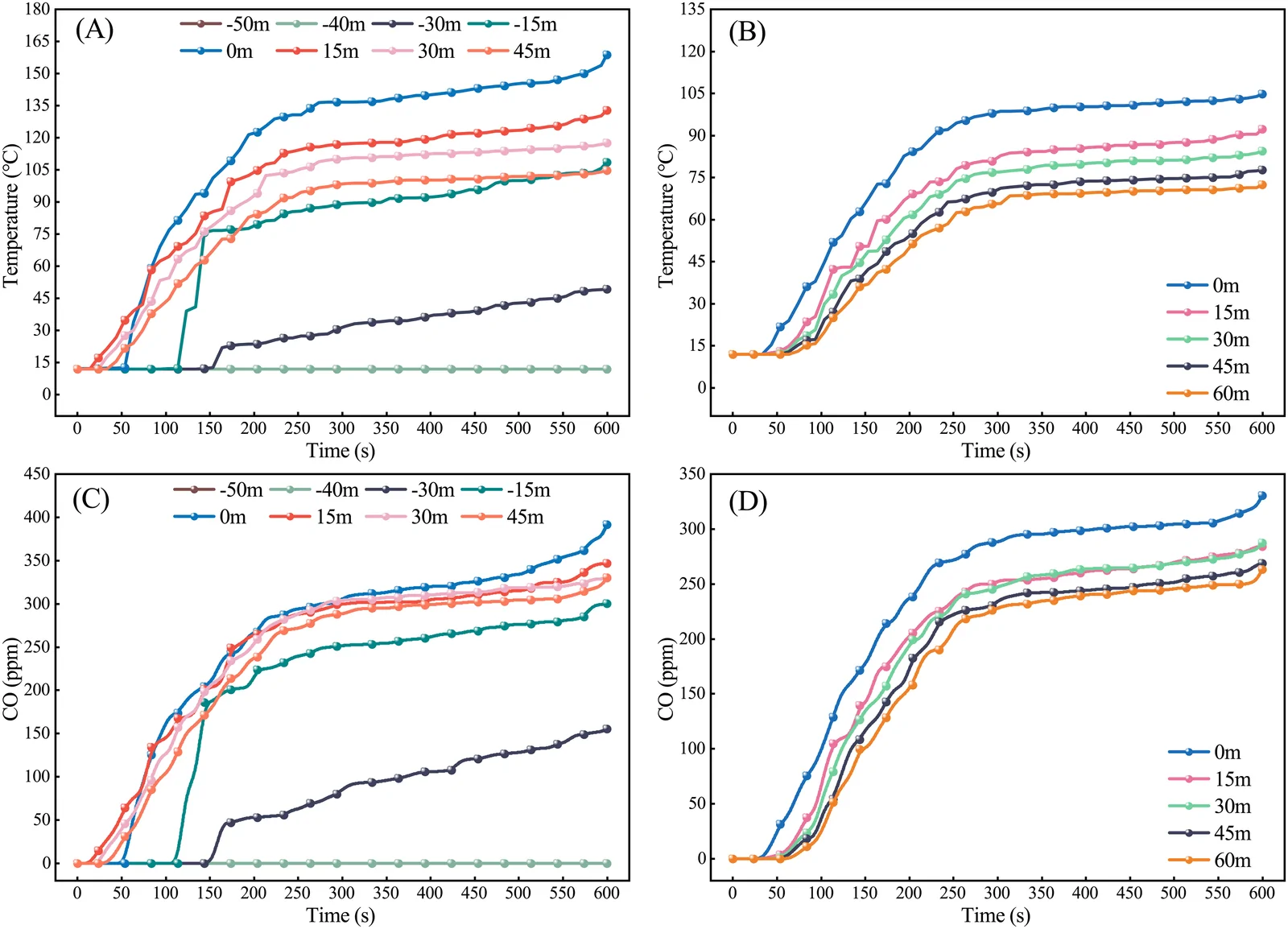
Temperature and CO concentration at representative points under 2.8 m/s ventilation. (A) Temperature in the belt roadway. (B) Temperature at the working face. (C) CO concentration in the belt roadway. (D) CO concentration at the working face.

Under the highest ventilation velocity of 3.2 m/s, near-source hot-smoke accumulation and upstream backflow were largely controlled. As shown in Fig 20, the temperature and CO concentration at the −50 m and −40 m monitoring points in the belt roadway remained close to their initial levels, while only a weak response was observed at the −30 m point during the later stage. The near-baseline responses at −50 and −40 m and the weak late response at −30 m confirm the strong suppression of remote upstream back-layering. At the 0 m monitoring point, the temperature and CO concentration at 600 s were approximately 140°C and 315 ppm, respectively, both lower than under the preceding ventilation conditions. Meanwhile, the CO concentration at the downstream 15 m monitoring point remained close to 340 ppm, and some downstream points exhibited higher concentrations than the near-fire point. This suggests that CO was transported downstream under forced airflow and accumulated along the downstream path. In the working-face region, the CO concentration at the 0 m monitoring point was approximately 310 ppm, with relatively small differences among the monitoring points. At 3.2 m/s, the weak upstream response contrasted with CO concentrations of approximately 340 ppm at 15 m downstream and 310 ppm at the working-face entrance, indicating that the region of pronounced CO response shifted downstream under strong longitudinal ventilation.

**Fig 20:**
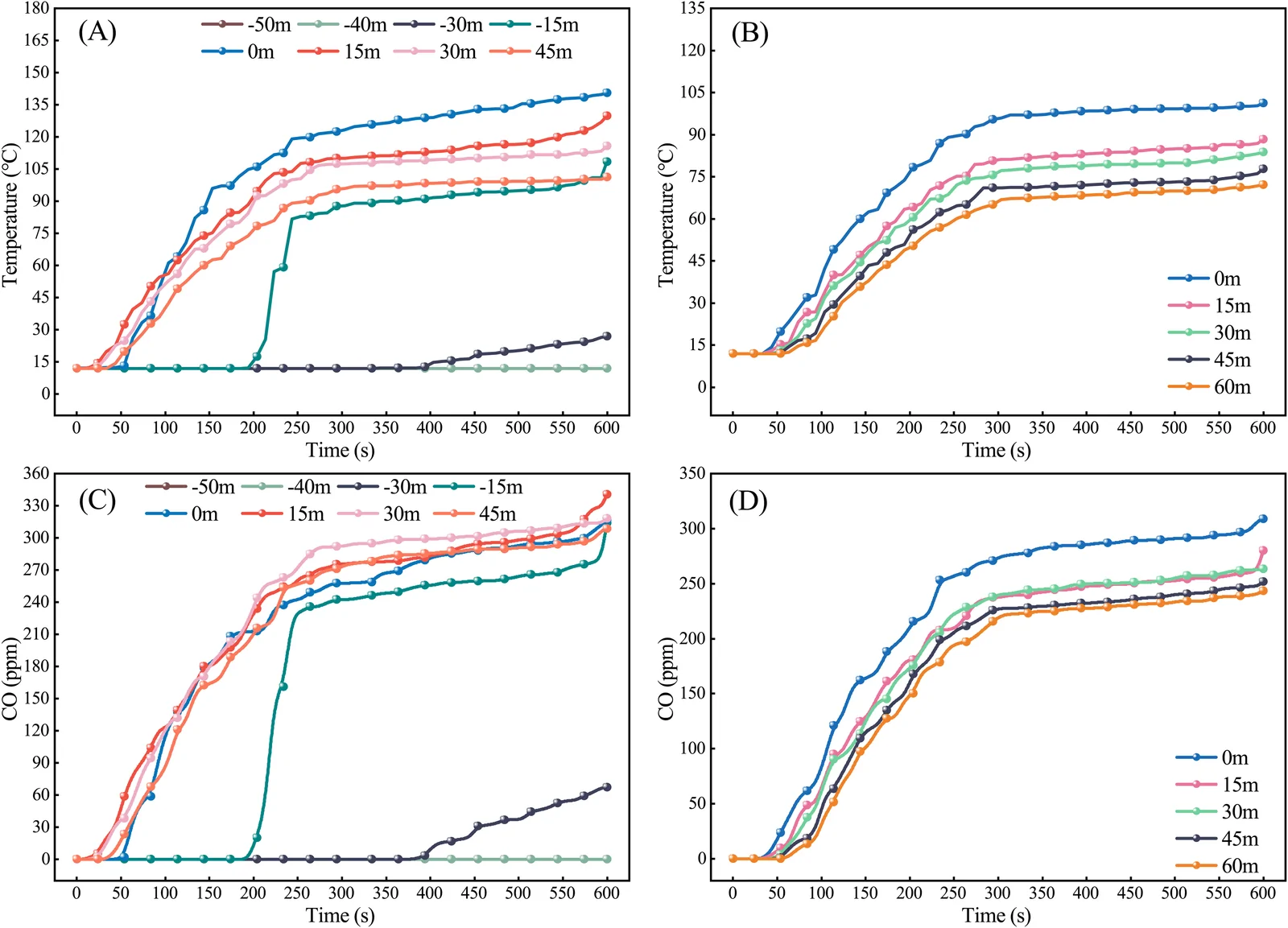
Temperature and CO concentration at representative points under 3.2 m/s ventilation. (A) Temperature in the belt roadway. (B) Temperature at the working face. (C) CO concentration in the belt roadway. (D) CO concentration at the working face.

Across Figs 14–20, increasing ventilation velocity delayed and weakened the remote-upstream response while concentrating earlier and more sustained responses along the downstream path. The change reflects a shift from buoyancy-supported roof-level back-layering to longitudinal-momentum-dominated transport. Temperature attenuated more rapidly than CO after the smoke entered the working face because cooler-air entrainment and turbulent mixing progressively reduced the thermal contrast of the smoke layer, whereas CO continued to migrate with the mixed smoke. The turning and connecting regions further redistributed momentum and produced local mixing, so the reduction in upstream response was accompanied by persistent downstream and working-face exposure. The peak values used to quantify these spatial differences are listed in Tables 4 and 5.

**Table 4:** Peak temperature at representative points under the 4800 kW thermal-source scenario (°C).

Air velocity (m/s)	Upstream 30 m	Upstream 15 m	Fire source	Downstream 15 m	Downstream 30 m	Working face 0 m	Working face 30 m	Working face 60 m
0.8	128.29	153.05	200.96	163.89	138.55	122.62	88.05	74.81
1.2	122.30	146.45	180.85	155.40	129.45	114.26	86.67	73.26
1.6	95.52	126.83	167.62	141.45	120.29	107.27	84.17	70.20
2.0	79.31	116.67	155.15	132.23	117.39	103.5	81.86	69.99
2.4	68.08	120.04	153.31	141.86	120.75	105.55	86.28	72.99
2.8	49.28	108.65	158.88	132.76	117.55	104.81	84.57	72.45
3.2	26.86	108.36	140.42	129.87	115.61	101.17	83.77	72.19

**Table 5:** Peak CO concentration at representative points under the 4800 kW thermal-source scenario (ppm).

Air velocity (m/s)	Upstream 30 m	Upstream 15 m	Fire source	Downstream 15 m	Downstream 30 m	Working face 0 m	Working face 30 m	Working face 60 m
0.8	488.68	511.18	570.29	490.17	460.47	465.04	409.67	366.87
1.2	460.94	473.25	502.13	442.67	406.85	412.65	357.08	341.50
1.6	334.31	376.05	436.38	377.61	354.41	352.81	296.98	273.91
2.0	252.97	318.01	364.78	349.57	322.38	312.79	279.70	268.94
2.4	220.69	343.37	377.3	374.1	344.45	333.05	291.29	271.32
2.8	155.36	300.32	391.86	346.85	330.03	330.39	287.25	263.52
3.2	67.37	313.67	316.63	340.45	318.22	308.57	263.09	243.52

At 0.8 m/s, the peak temperature and CO concentration 30 m upstream were 128.29°C and 488.68 ppm, respectively, indicating long-distance upstream transport. At 3.2 m/s, these values decreased to 26.86°C and 67.37 ppm, whereas the corresponding peaks at 15 m downstream remained 129.87°C and 340.45 ppm and those at the working-face entrance remained 101.17°C and 308.57 ppm. The remote-upstream response was therefore more sensitive to ventilation than the downstream and working-face responses. At 3.2 m/s, the downstream 15 m CO peak exceeded the source-point peak because the maxima occurred at different times, indicating a downstream shift in the location of the strongest CO response rather than a simultaneous concentration inversion.

Along the working face, temperature decreased more rapidly than CO. At 0.8 m/s, the temperature peak decreased from 122.62 to 74.81°C between 0 and 60 m, while the CO peak decreased from 465.04 to 366.87 ppm. This difference reflects the stronger influence of cooler-air entrainment and turbulent mixing on the temperature field, whereas CO remained coupled to the downstream mixed-smoke flow.

To further reveal the influence of ventilation velocity on smoke-transport intensity, a dimensionless comparative analysis was conducted using the peak temperature and CO concentration at fixed monitoring points under the 4800 kW thermal-source scenario. Because the monitoring locations and baseline peak levels differed, the 0.8 m/s case was selected as the reference, and the peak values at each point were normalized. Ventilation velocity was expressed in the Froude-type dimensionless form $\begin{array}{c} \left(V^{*}=u/\sqrt{gH}\right) \end{array}$. For comparison among the simulated cases, the peak temperature rise and peak CO concentration at each monitoring point were normalized by their corresponding values under the 0.8 m/s reference condition, as defined in Eqs. (18)–(20).


$$\begin{array}{c} V^{*}=\frac{u}{\sqrt{gH}} \end{array}$$ (18)

$$\begin{array}{c} \theta_{i}=\frac{T_{i,max}-T_{\text{0}}}{T_{i,max,0\mathrm{.8}}-T_{\text{0}}} \end{array}$$ (19)

$$\begin{array}{c} C_{i}^{*}=\frac{C_{i,max}}{C_{i,max,0\mathrm{.8}}} \end{array}$$ (20)

Where $V^{*}$ is the dimensionless roadway air velocity; u is the roadway air velocity, m/s; g is the gravitational acceleration, taken as 9.81m/s^2^; H is the roadway height, taken as 5 m in this study; $\theta_{i}$ is the dimensionless temperature rise at the i-th fixed monitoring point; $C_{i}^{*}$ is the dimensionless CO concentration at the i-th fixed monitoring point;$T_{i,max}$ and $C_{i,max}$ are the peak temperature and peak CO concentration, respectively, at the i-th fixed monitoring point under different air velocities; $T_{i,max,0\mathrm{.8}}$ and $C_{i,max,0\mathrm{.8}}$ are the corresponding peak temperature and peak CO concentration at the same monitoring point under the 0.8 m/s condition. The initial model temperature was 285.15 K (approximately 12°C). The dimensionless peak temperature rise and CO concentration at representative monitoring points are listed in Tables 6 and 7, respectively.

**Table 6:** Dimensionless peak temperature rise at representative points under the 4800 kW thermal-source scenario.

Air velocity (m/s)	$V^{*}$	Upstream 30 m	Upstream 15 m	Fire source	Downstream 15 m	Downstream 30 m	Working face 0 m	Working face 30 m	Working face 60 m
0.8	0.114	1	1	1	1	1	1	1	1
1.2	0.171	0.948	0.953	0.894	0.944	0.928	0.924	0.982	0.975
1.6	0.228	0.718	0.814	0.824	0.852	0.856	0.861	0.949	0.927
2.0	0.286	0.579	0.742	0.758	0.792	0.833	0.827	0.919	0.923
2.4	0.343	0.482	0.766	0.748	0.855	0.859	0.846	0.977	0.971
2.8	0.400	0.321	0.685	0.777	0.795	0.834	0.839	0.954	0.962
3.2	0.457	0.128	0.683	0.680	0.776	0.819	0.806	0.944	0.958

**Table 7:** Dimensionless peak CO concentration at representative points under the 4800 kW thermal-source scenario.

Air velocity (m/s)	$V^{*}$	Upstream 30 m	Upstream 15 m	Fire source	Downstream 15 m	Downstream 30 m	Working face 0 m	Working face 30 m	Working face 60 m
0.8	0.114	1	1	1	1	1	1	1	1
1.2	0.171	0.943	0.926	0.880	0.903	0.884	0.887	0.872	0.931
1.6	0.228	0.684	0.736	0.765	0.77	0.77	0.759	0.725	0.747
2.0	0.286	0.518	0.622	0.640	0.713	0.700	0.673	0.683	0.733
2.4	0.343	0.452	0.672	0.662	0.763	0.748	0.716	0.711	0.740
2.8	0.400	0.318	0.588	0.687	0.708	0.717	0.710	0.701	0.718
3.2	0.457	0.138	0.614	0.555	0.695	0.691	0.664	0.642	0.664

As shown in Tables 6 and 7, the dimensionless temperature rise and dimensionless CO concentration at different monitoring points exhibited pronounced spatial differences with variations in roadway air velocity. The most significant attenuation occurred at the 30 m upstream monitoring point, where the dimensionless temperature rise decreased from 1.000 under the 0.8 m/s condition to 0.128 under the 3.2 m/s condition, while the dimensionless CO concentration decreased from 1.000 to 0.138. This indicates that the far-upstream region was the most sensitive to changes in roadway air velocity and can effectively reflect variations in the intensity of long-distance smoke backflow. The 15 m upstream monitoring point was located closer to the fire source. Although its dimensionless temperature rise and dimensionless CO concentration generally decreased with increasing air velocity, they remained at certain levels under the 2.4 m/s and higher velocity conditions. This suggests that the near-fire upstream region was still affected by the buoyant fire plume, local entrainment, and near-source disturbances. To characterize the combined thermal and toxic-gas responses at the monitoring points, a composite smoke response index was defined as the arithmetic mean of the dimensionless peak temperature rise and dimensionless peak CO concentration. At the upstream monitoring points, the index characterizes the combined response associated with smoke back-layering; at the downstream monitoring points, it characterizes the combined response associated with smoke transport along the longitudinal airflow. The index is expressed as follows:


$$\begin{array}{c} I_{b,s,j}=\frac{\theta_{s,j}+C_{s,j}^{*}}{\text{2}} \end{array}$$ (21)

Where $I_{b,s,j}$ is the integrated smoke response index at position j on side s; s = u and s = d denote the upstream and downstream sides of the fire source, respectively; j corresponds to 15 m and 30 m; $\theta_{s,j}$is the dimensionless peak temperature rise at the corresponding monitoring point; and $C_{s,j}^{*}$ is the dimensionless peak CO concentration at the corresponding monitoring point. A larger $I_{b,s,j}$value indicates that the combined smoke response at the monitoring point is closer to that under the reference condition of 0.8 m/s. For upstream monitoring points, a decrease in $I_{b,u,j}$ indicates the suppression of smoke backflow; for downstream monitoring points, a decrease in $I_{b,d,j}$ indicates the attenuation of downstream smoke transport.

Using the dimensionless data at the 15 and 30 m monitoring points upstream and downstream of the fire source in Tables 6 and 7, the relationships between the composite smoke response index and dimensionless ventilation velocity were established, as shown in Fig 21.

**Fig 21:**
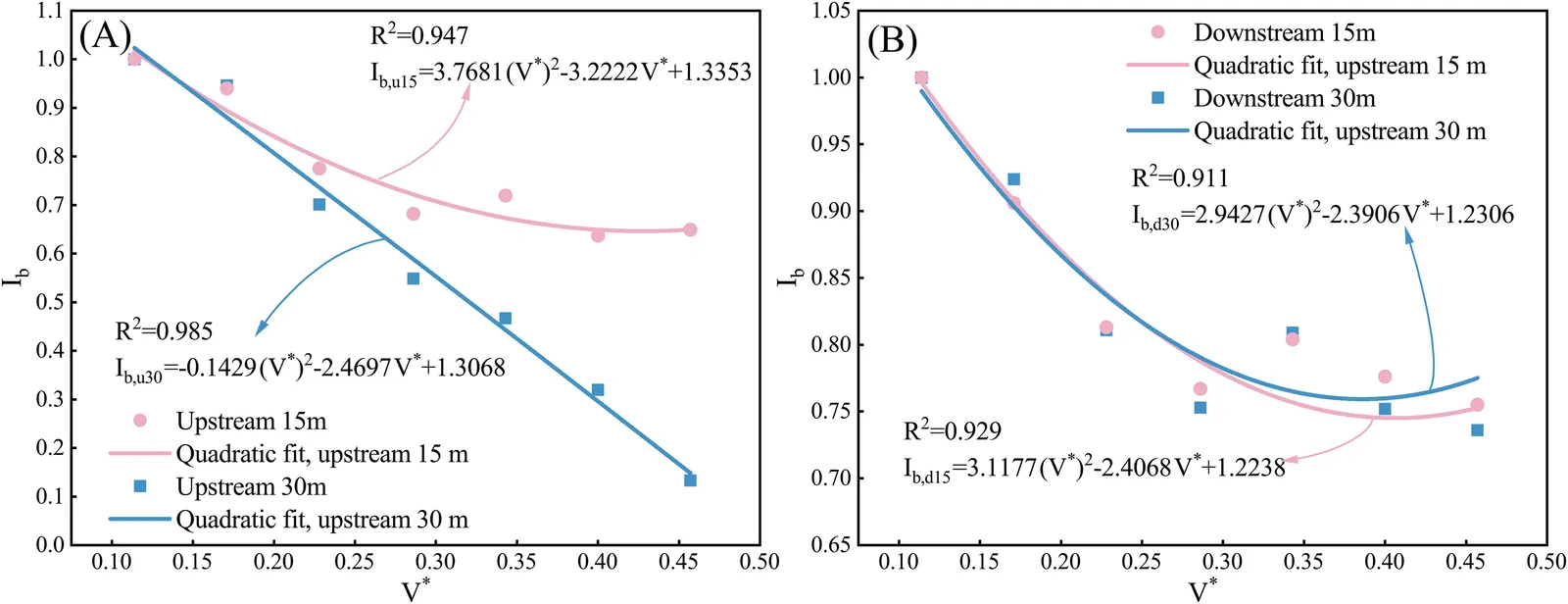
Composite smoke response index versus dimensionless ventilation velocity under the 4800 kW thermal-source scenario. (A) Upstream fitted curves. (B) Downstream fitted curves.

As shown in Fig 21, the composite smoke response indices on the upstream and downstream sides exhibited different trends with increasing dimensionless ventilation velocity. For the 30 m upstream monitoring point, regression of *I*_*b,u30*_ against V* yielded a coefficient of determination of R^2^ = 0.985, indicating that the fitted curve adequately described the simulated response. By contrast, although *I*_*b,u15*_ generally decreased, it remained at a certain level under medium-to-high velocity conditions because the near-fire upstream region was still affected by fire-plume inclination, local entrainment, and turbulent disturbances. The downstream indices *I*_*b,d30*_ and *I*_*b,d15*_ showed smaller overall reductions and a local rebound near 2.4 m/s. These contrasting trends indicate that increasing ventilation progressively suppressed upstream smoke back-layering while redirecting hot smoke and CO along the downstream flow path.

The 30 m upstream composite response index I_b,u30_ exhibited the most pronounced decrease with increasing ventilation velocity and was therefore selected to characterize the transition in the remote-upstream smoke response. For consistent quantitative characterization of the response transition, the midpoint of the normalized response, I_b,u30_ = 0.5, was adopted as the transition criterion. With the 0.8 m/s case used as the reference, this criterion corresponds to a 50% reduction in the combined peak temperature-rise and CO response.

## Conclusions

This study combined small-scale combustion experiments using PVG1250S conveyor-belt specimens with PyroSim numerical simulations to investigate fixed-point temperature and CO/CO_2_ responses under different ventilation velocities and smoke transport in a U-shaped belt-conveyor roadway under prescribed thermal-source conditions. The main conclusions are as follows.

(1) As the ventilation velocity increased from 0.4 to 1.6 m/s, the peak CO concentration at the monitoring point decreased from 453 to 175 ppm, whereas the peak flow-rate-corrected CO transport index increased by 54.43%. This divergence indicates that a lower fixed-point concentration can coexist with stronger downstream convective transport. The fixed-point temperature response was non-monotonic with ventilation velocity. The upper-layer temperature exceeded the lower-layer temperature in all cases, and the maximum simultaneous vertical temperature difference ranged from 51.14 to 59.96°C, confirming persistent upper-layer smoke transport and vertical thermal stratification. Both toxic-gas transport and the thermal response of the upper hot-smoke layer should therefore be considered when characterizing the effects of ventilation.
(2) The numerical simulations characterized the spatial evolution of smoke within the U-shaped roadway under the combined effects of fire-induced buoyancy and longitudinal ventilation. At low ventilation velocities, buoyancy dominated smoke movement, causing high-temperature smoke to spread upstream beneath the roof and leading to localized accumulation of heat and CO near the fire source. As the ventilation velocity increased, the longitudinal airflow increasingly suppressed and entrained the fire plume, thereby weakening upstream smoke back-layering. Consequently, smoke transport gradually shifted from buoyancy-dominated to ventilation-dominated behavior. Successive turning through the working face and connecting roadway was accompanied by momentum redistribution, local recirculation, and shear entrainment, together with smoke bifurcation, local accumulation, and continued downstream transport. The region of pronounced smoke response progressively extended from the source and upstream roadway toward the working face, connecting roadway, and return-air roadway as ventilation increased.
(3) Under the prescribed 4800 kW thermal-source condition, the composite smoke-response index at 30 m upstream decreased continuously with increasing ventilation velocity, and the fitted relationship yielded R^2^ = 0.985. The marked attenuation of the remote-upstream response occurred mainly within 2.0–2.4 m/s, with a scenario-specific characteristic transition velocity of approximately 2.24 m/s. Near this range, remote upstream back-layering was markedly weakened, whereas temperature and CO responses persisted downstream and at the working face. This reflects a trade-off between suppressing upstream smoke back-layering and maintaining downstream smoke transport.

## Supporting information

S1 Data Raw data for fig 1-21. (XLSX)

## Supporting information

S1 Data Data underlying the experimental measurements, numerical simulation results, and dimensionless analyses. This file contains the source data used to generate Figs 2–6 and 14–21.
(XLSX)
